# Supplementary material for: Mini-batch optimization enables training of ODE models on large-scale datasets
Source: Nat Commun. 2022 Jan 10;13:34. doi: 10.1038/s41467-021-27374-6 (PMC8748893; doi:10.1038/s41467-021-27374-6)
Supplement: Supplementary file 1 — Supplementary Information [file 41467_2021_27374_MOESM1_ESM.pdf]

Supplementary information to mini-batch optimization enables training of ODE models on large-scale datasets

Paul Stapor [1, 2], Leonard Schmiester [1, 2], Christoph Wierling [3], Simon Merkt [4], Dilan Pathirana [4], Bodo M.H. Lange [3], Daniel Weindl [1], and Jan Hasenauer [1, 2, 4, *]

[1] Helmholtz Zentrum München - German Research Center for Environmental Health, Institute of Computational Biology, 85764 Neuherberg, Germany,

[2] Technische Universität München, Center for Mathematics, Chair of Mathematical Modeling of Biological Systems, 85748 Garching, Germany,

[3] Alacris Theranostics GmbH, 12489 Berlin, Germany, and

[4] Universität Bonn, Faculty of Mathematics and Natural Sciences, 53115 Bonn, Germany.

June 2021

In this Supplementary Information, we provide a detailed description of the employed methods and algorithms, which were employed in our studies on mini-batch optimization. Therefore, the sections on "Parameter estimation for ordinary differential equation models", "Mini-batch optimization", and "Enhancements of mini-batch optimization for ODE models" complement the Methods section. The Supplementary Information also provides a section on "Proliferation output of the large-scale pan-cancer model" and the "Interpretation and discussion of the in-silico knockout study", where we also discuss possible further improvements. This complements the Dicsussion section.

1 Parameter estimation for ordinary differential equation models

Modeling of chemical reaction networks with ordinary differential equations (ODEs)

Ordinary differential equations (ODEs) are a common approach to model the time-evolution of bio-chemical species. The underlying assumptions when using ODE models are first, that spatial inhomogeneities can be neglected within one compartment and second, that all species are abundant enough that the process is governed by deterministic dynamics, in contrast to stochastic dynamics, which can be found in systems with low copy numbers.

In most applications, an ODE model describes the time evolution of a state vector $x(t) \in \mathbb{R}^{n_x}$ of concentrations of $n_x \in \mathbb{N}$ biochemical species, which are, for example, (phospho-)proteins or mRNA levels, at time $t \in [0, T]$. The time evolution of x is given by a vector field f, which depends on unknown parameters $\theta \in \mathbb{R}^{n_\theta}$ and known input parameters $u \in \mathbb{R}^{n_u}$:

$$\frac{d}{dt}x(t,\theta,u) = f(x(t,\theta,u),\theta,u), \quad \text{with } x(0) = x_0(\theta,u) \tag{1}$$

*To whom correspondence should be addressed.

Parameters are typically reaction rates constants or initial concentrations, which can not be measured. Input parameters are often external stimuli such as treatments with drugs or other ligands, or measurable initial concentrations, mRNA expression levels or genetic profiles. Depending on f, θ, and u, a solution of the ODE given in Equation (1) may or may not exist for a time interval $[0, T]$. However, in most of the considered cases, it exists, but is not available in closed form. This makes it necessary to use numerical solvers to integrate/solve the ODE. Again depending on the equation, the employed ODE solver, and its settings, a numerical integration of Equation (1) may fail, which is a common problem when using ODE models of biological process, at least during parameter estimation (7; 8). Hence, involved implementations of, e.g., implicit Runge-Kutta, Adams-Moulton, or BDF schemes are used, which allow adaptive time-stepping and automated error control. This reduces integration failure and to ensure the desired accuracy of the computed results (7; 8; 11).

An ODE model also allows the description of the time-evolution of a vector of measurable quantities $y(t) \in \mathbb{R}^{n_y}$, which we will call observables. In realistic modeling applications, it is often impossible to measure state variables directly, as – in many cases – measurement techniques only allow the assessment on a relative scale (e.g., quantitative PCR), maybe with an additional offset (e.g., immunoblotting), on a (bi-)exponential scale (e.g., flow cytometry), or as sums of certain state-variables (e.g., if an antibody binds to a protein, independent of its phosphorylation state). For this reason, we introduce observable functions:

$$y(t, \theta, u) = h(x(t, \theta, u), \theta) \tag{2}$$

An observable can be matched to experimental data D, taken at timepoints $t_i, i = 1, \dots, n_t$ and measured for n_e experimental conditions. Distinct experimental conditions differ through the vectors of input parameters $u_k \in \mathbb{R}^{n_u}$, $k = 1, \dots, n_e$, which describe all the differences in the model, which are necessary to capture different experimental setups, such as drug treatments or other external stimuli, growth conditions for bacteria or differences in, e.g., mRNA expression levels for different cell-lines. In most cases, u determines the initial values of the ODE, but it can also comprise a time-resolved inputs, which can be modelled using splines. To simulate the whole dataset D once, n_e different initial value problems have to be solved. D is then given as:

$$D = \{\bar{y}_{ij}^k\}_{\substack{i=1,\dots,n_y^k \\ j=1,\dots,n_t}}^{k=1,\dots,n_e} \tag{3}$$

Experimental data are noise-corrupted, and thus we have to choose a noise model. In this study, we follow the most common approach and assume additive Gaussian noise, yielding:

$$\bar{y}_{ij}^k = y_i(t_j, \theta, u^k) + \varepsilon_{ij}^k, \qquad \text{with } \varepsilon \sim \mathcal{N}\left(0, \left(\sigma_{ij}^k\right)^2\right) \tag{4}$$

Here, σ_{ij}^k denotes the standard deviations for the respective timepoints. This statistical model allows us to compute the likelihood of a measurement value $y_i(t_k, \theta, u^k)$ given a parameter vector θ, assuming independence of the measurement noise terms:

$$\mathcal{L}(D \,|\, \theta) = \prod_{k=1}^{n_e} \prod_{i=1}^{n_y^k} \prod_{j=1}^{n_t} \frac{1}{\sqrt{2\pi}\sigma_{ij}^k} \exp\left\{ -\frac{1}{2} \left(\frac{\bar{y}_{ij}^k - y_i(t_k, \theta, u^k)}{\sigma_{ij}^k} \right)^2 \right\} \tag{5}$$

Parameter estimation, local, and global optimization

The statistical model makes it possible to fit the unknown model parameters to the measurement data, by maximizing the likelihood $\mathcal{L}(D \,|\, \theta)$. However, it is more common to work with the negative logarithm of the likelihood, as first, most optimization algorithms are implemented as minimization algorithms and second, the logarithm of the likelihood tends to be more convex and is hence numerically better tractable (11). This

yields the negative log-likelihood as objective or cost function:

$$J(\theta) = -\log\left(\mathcal{L}(D \mid \theta)\right) = \frac{1}{2}\sum_{k=1}^{n_e}\sum_{i=1}^{n_y^k}\sum_{j=1}^{n_t}\left(\left(\frac{\bar{y}_{ij}^k - y_i(t_k,\theta,u^k)}{\sigma_{ij}^k}\right)^2 + \log\left(2\pi\left(\sigma_{ij}^k\right)^2\right)\right) \tag{6}$$

For additive Gaussian noise with known measurement noise σ_{ij}^k – as assumed in this study – the logarithmic term is just a constant, offsetting the objective function value from 0, but not affecting the location of the minimum. In this case, minimizing this objective function is equivalent to using a weighted least squares algorithm. In parameter estimation/optimization, the aim is to find the global minimizer of J, which is the maximum likelihood estimator θ^{MLE} of $\mathcal{L}(D \mid \theta)$:

$$\theta^{MLE} = \text{argmin}_{\theta\in\Omega}\, J(\theta) \tag{7}$$

Hence, minimizing J is equivalent to optimizing the quality of the fit of the model to the data. Usually, the parameter vector θ is restricted to a region $\Omega \in \mathbb{R}^{n_\theta}$ in parameter space, which is assumed to be biologically plausible.

For global optimization of θ, many methods exist (15). A simple idea, called multi-start local optimization, is to run algorithms which are guaranteed to find a local minimum from many random initial points (11; 20). These results can be sorted by the final objective function values and visualized in waterfall plots, which can – if optimization worked well – reveal the structure of the local minima of the objective function. The best local optimum is then assumed to be the global optimum. Other common methods to find global optima are genetic or swarm-based algorithms, simulated annealing, or scatter-search, which combines genetic algorithms with multi-start local optimization. For ODE models however, it is well established that multi-start local optimization and scatter-search outperform other approaches for most, though maybe not for all, cases (11; 15; 20).

Local optimization algorithms usually exploit derivative information, such as the gradient of the objective function. There are three common methods of gradient computation for ODE models: finite difference schemes, forward sensitivity analysis, and adjoint sensitivity analysis. For large-scale ODE models with many parameters, adjoint sensitivity analysis (2) has been shown to be the most efficient approach and subsequently for local optimization (2; 3; 20). Different local optimization algorithms exist, among which gradient descent, quasi-Newton methods such as (L-)BFGS, Gauss-Newton, trust-region approaches such as the dogleg method, or interior-point methods are the most popular. For parameter estimation of very large ODE models, implementations of interior-point methods combined with (L-)BFGS schemes and either trust-region or line-search methods for controlling the step size have shown their effectiveness (2; 3; 16; 20).

2 Mini-batch optimization

Mini-batch optimization is a particular type of local optimization and only capable of finding local optima (13). The basic idea consists of exploiting the fact that the objective function and its gradient have the structure of a sum:

$$J(\theta) = \frac{1}{2}\sum_{k=1}^{n_e}\underbrace{\sum_{i=1}^{n_y^k}\sum_{j=1}^{n_t}\left(\left(\frac{\bar{y}_{ij}^k - y_i(t_k,\theta,u^k)}{\sigma_{ij}^k}\right)^2 + \log\left(2\pi\left(\sigma_{ij}^k\right)^2\right)\right)}_{=J^k(\theta)} = \sum_{k=1}^{n_e} J^k(\theta) \tag{8}$$

$$\nabla_\theta J(\theta) = \ldots = \sum_{k=1}^{n_e}\nabla_\theta J^k(\theta) \tag{9}$$

In each optimization step r, a random subset – the mini-batch – $S_r \subseteq \{1, \ldots, n_e\}$ is chosen, and the objective function and its gradient are computed only based on S_r. It is important to note that no experimental condition is chosen twice until the full dataset has been evaluated, a time-frame which is called one epoch. Hence, the mini-batches belonging to one epoch are disjoint. Theoretically, also the inner sum structure (over the indices i and j) could be used for mini-batching. However, when working with ODE models, the computational demanding part is the solution of the initial value problem for an experimental condition k. Once this is done, evaluating the different observables y_i at different time points t_j happens at negligible cost. Hence, for most applications on ODE models, mini-batching will only make sense over the conditions k.

Mini-batch optimization algorithms in a nutshell

The way how the parameter update is executed, i.e., how $\theta^{(r+1)}$ is computed from $\theta^{(r)}$ and the gradient (estimate) $\sum_{k \in S_r} \nabla_\theta J_k(\theta^{(r)})$, depends on the chosen algorithm. Some of the most commonly used algorithms which we investigated in our study are:

- Stochastic gradient descent (SGD) (13), sometimes called "Vanilla SGD", which is the simplest possible algorithm, using only the negative gradient of the objective function as update direction (Supplementary Information, Algorithm 1).
- Stochastic gradient descent with momentum (10; 17), a common variant, which uses a decaying average of negative gradients as direction instead of the negative objective function gradient alone (Supplementary Information, Algorithm 2).
- RMSProp (18), a so-called adaptive algorithm, which rescales the current gradient by a decaying average over root-mean-squares over the previous objective function gradients (Supplementary Information, Algorithm 3).
- Adam (6), another adaptive algorithm, which attempts to combine the benefits of RMSProp with the momentum approach by using two decaying averages (Supplementary Information, Algorithm 4).

A good summary of these and further mini-batch optimization algorithms can be found in (4).

As Adam is a very popular algorithm and has more tuning parameters than the other algorithms, we investigated two different settings of these tuning parameters: The two decaying averages are governed by two coefficients, usually denoted as ρ_1 and ρ_2, averaging the gradient and its norm, respectively. The original publication sets the former to a value of 0.9 and the latter to 0.999, which we denoted as standard version of Adam. We also investigated a simplified/tuned version, in which we set $\rho_1 = \rho_2 = 0.9$.

Learning rates and step-sizes during optimization

Despite their different approaches, all the mentioned mini-batch algorithms have some features in common. First, they are not guaranteed to – and, in fact, don't even try to – produce a series of parameter vectors, along which the objective function decreases monotonically. Hence, it is to be expected that the progress which is made in one optimization step can be (at least partly) undone in another, subsequent step. Over the whole optimization process however, most of them are guaranteed to converge to a local minimum in a probabilistic sense. This is a striking difference to most local full-batch optimization algorithms, which at least try to or can even guarantee to produce monotonically decreasing trajectories of objective function values. Second, all of these mini-batch optimization algorithms rescale the proposed optimization step with a factor η, called the learning rate. If we assume a given algorithm in optimization step r to produce a

Algorithm 1 Parameter update for stochastic gradient descent

Initialization:

Set initial learning rate η_0
Set final learning rate η_N
Set number of epochs N

Procedure for parameter update during optimization:

Get index of current epoch k
Get current parameter vector: θ
Get gradient estimate for current mini-batch: g
Compute update direction (normalized negative gradient estimate): $\delta \leftarrow -g/\|g\|$
Compute current learning rate: $\eta \leftarrow \eta(k, \eta_0, \eta_N, N)$
Update parameter vector: $\theta \leftarrow \theta + \eta \cdot \delta$

Algorithm 2 Parameter update for stochastic gradient descent with momentum

Initialization:

Set initial learning rate η_0
Set final learning rate η_N
Set number of epochs N
Set decay rate for momentum $\alpha = 0.8$
Set momentum vector $v = 0 \in \mathbb{R}^{n_\theta}$

Procedure for parameter update during optimization:

Get index of current epoch k
Get current parameter vector: θ
Get gradient estimate for current mini-batch: g
Get momentum vector: v
Compute new momentum: $v \leftarrow \alpha v + (1-\alpha)g$
Compute update direction: $\delta \leftarrow -v/\max(\|v\|, 1)$
Compute current learning rate: $\eta \leftarrow \eta(k, \eta_0, \eta_N, N)$
Update parameter vector: $\theta \leftarrow \theta + \eta \cdot \delta$

Algorithm 3 Parameter update step for RMSProp

Initialization:

Set initial learning rate η_0
Set final learning rate η_N
Set number of epochs N
Set decay rate for gradient norm history $\rho = 0.9$
Set gradient norm history vector $h = 0 \in \mathbb{R}^{n_\theta}$
Set stabilization constant $\varepsilon = 10^{-7}$

Procedure for parameter update during optimization:

Get index of current epoch k
Get current parameter vector: θ
Get gradient estimate for current mini-batch: g
Get gradient norm history vector: h
Compute new gradient norm history vector (using element-wise multiplication): $h \leftarrow \rho h + (1-\rho) g \odot g$
Compute update direction (using element-wise division): $\delta \leftarrow -g \oslash (\sqrt{h} + \varepsilon)$
Compute current learning rate: $\eta \leftarrow \eta(k, \eta_0, \eta_N, N)$
Update parameter vector: $\theta \leftarrow \theta + \eta \cdot \delta$

Algorithm 4 Parameter update step for Adam

Initialization:

Set initial learning rate η_0
Set final learning rate η_N
Set number of epochs N
Set decay rate for gradient history $\rho_1 = 0.9$
Set decay rate for gradient norm history $\rho_2 = 0.999$ (or $\rho_2 = 0.9$ for tuned version)
Set gradient history vector $h_1 = 0 \in \mathbb{R}^{n_\theta}$
Set gradient norm history vector $h_2 = 0 \in \mathbb{R}^{n_\theta}$
Set stabilization constant $\varepsilon = 10^{-7}$

Procedure for parameter update during optimization:

Require: Index of current optimization step ℓ
Get index of current epoch k
Get current parameter vector: θ
Get gradient estimate for current mini-batch: g
Get gradient history vector: h_1
Get gradient norm history vector: h_2
Compute new gradient history vector: $h_1 \leftarrow \rho_1 h_1 + (1 - \rho_1) g$
Compute new gradient norm history vector: $h_2 \leftarrow \rho_2 h_2 + (1 - \rho_2) g \odot g$
Compute update direction (using element-wise division, applying bias-correction):
$\delta \leftarrow -\frac{h_1}{1-\rho_1^\ell} \oslash \left(\sqrt{\frac{h_2}{1-\rho_2^\ell}} + \varepsilon \right)$
Compute current learning rate: $\eta \leftarrow \eta(k, \eta_0, \eta_N, N)$
Update parameter vector: $\theta \leftarrow \theta + \eta \cdot \delta$

parameter update (direction) δ_r, then the next proposed parameter vector will be

$$\theta^{(r+1)} = \theta^{(r)} + \eta_r \cdot \delta_r, \tag{10}$$

with η_r being the learning rate at step r. Obviously, η affects the step size of the optimizer. But as for most algorithms $\|\delta_r\| \neq 1$, it is not identical to the step size. In many publications, the learning rate is either fixed to a previously chosen value, such as 10^{-3} (14), which is a commonly used value for training deep neural nets, or prescheduled over the optimization process (4). In our studies on the small- to medium-scale models, we worked in total with four different learning rate schedules:

- Schedule 1: A high learning rate, which logarithmically decreased from 10^0 to 10^{-3}.
- Schedule 2: A medium learning rate, which logarithmically decreased from 10^{-1} to 10^{-4}.
- Schedule 3: A low learning, which logarithmically decreased from 10^{-2} to 10^{-5}.
- Schedule 4: A constant learning rate, fixed to the value 10^{-3}.

We assumed that the crucial quantity for the optimization process is the actual optimizer step-size and not the learning rate. From our experience with parameter estimation of ODE models, we aimed at initial optimizer step-sizes to be roughly in the interval $[1, 10]$, rather tending towards 1 for smaller and rather tending towards 10 for larger models. It is important to note that this is a mere rule-of-thumb based on personal experience. However, this implies that different learning rates had to be chosen for the different models, since for Adam,

the initial step size is given by the following computation:

$$\|\Delta\theta\| = \|\eta\delta\| = \sqrt{\sum_{j=1}^{n_\theta} \frac{\frac{(1-\rho_1)g_j}{1-\rho_1^1}}{\sqrt{\frac{(1-\rho_2)g_j^2}{1-\rho_2^1}} + \varepsilon}} = \sqrt{\sum_{j=1}^{n_\theta} \frac{g_j}{g_j + \varepsilon}} \approx \sqrt{n_\theta} \tag{11}$$

Here, we used the notation from Algorithm 6. As we achieved best results for Schedule 2, i.e., medium learning rates on the small- and medium-scale models, we aimed at similar optimizer step-sizes on the large-scale model. This implied that we had to reduce the learning rates by an order of magnitude, as the large-scale model has about two order of magnitude more parameters than the small- and medium-scale models. For this reason, we used the two following learning rates with the two following names for the large-scale model:

- High learning rate, logarithmically decreasing from 10^{-1} to 10^{-4}.
- Low learning rate, logarithmically decreasing from 10^{-2} to 10^{-4}.

3 Enhancements of mini-batch optimization for ODE models

Motivation from established methods used in full-batch optimization of ODE models

Classic full-batch local optimization algorithms, which are used for parameter estimation of ODE models, employ trust-region or line-search methods for adaptive step-sizing (5; 12). These approaches provide an intuitive way of dealing with integration failure of the underlying ODE: If the ODE can not be solved, the objective function value and its gradient are set to infinity. This makes the proposed step unacceptable and leads to a shrinkage of the step-size, until the ODE can be solved again. In mini-batch optimization, similar approaches do not exist, which complicates a direct method transfer to parameter optimization of ODE models. Hence, we introduced two functionalities to overcome this issue. In both of them, we adapt the current learning rate, by multiplying it with two reduction factors β and γ, which mimic the role of a one-dimensional trust-region and a line-search, respectively.

Rescue interceptor to avoid ODE integration failure

The rescue interceptor is activated, if ODE integration (with gradient computation) fails: It undoes the last optimization step (but keeps the current mini-batch), multiplies the reduction factor β by r (in our implementation, we set $r = 0.2$, but any number $r < 1$ would do), and proposes a new step, for which the objective function and its gradient are computed. This procedure is repeated at most ten times, until either a parameter vector is found for which ODE integration is possible, or the local optimization run is finally stopped. Whenever ODE integration is successful, the reduction factor is mildly increased again by multiplication with c (in our case: $c = 1.3$, but any number $c > 1$ would do), to at most 1. Hence, the rescue interceptor mimics the behaviour of a trust-region implementation, by shrinking the trust-region whenever ODE integration failure is encountered, and expands the trust-region only slowly afterwards. Heuristically, this makes sense, as ODE integration would happen if the optimizer is in a region of the parameter space where the underlying ODE is particularly stiff. As one cannot assume to leave this region with only one optimizer step, the step-size is kept small for a longer period of time. In our implementation, it takes about six steps until the old step-size is recovered after ODE-integration failure. A detailed pseudo-code of this functionality is given in Algorithm 5.

Line-search functionality to improve optimization for high learning rates

The additional line-search works independently of the rescue interceptor and is based on the interpolation method, which is described in Chapter 3 of (9). After a parameter update is proposed, the objective function is evaluated without gradient – for the new parameter vector, but on the same mini-batch – and checked for improvement. If the new step yields an improvement, it is accepted, otherwise a backtracking line-search is performed according to the mentioned algorithm, by shrinking the reduction factor γ. However, once an optimization step is accepted, the reduction factor γ is set to 1. Hence, unlike the rescue interceptor, the line-search only affects the current optimization step, but not subsequent ones. A comparison of this approach to standard mini-batch optimization is given in Fig. 7 of the main manuscript, a detailed pseudo-code is provided in Algorithm 6.

Rescue interceptor and line-search can be combined, as they are not redundant. This can be seen in the following way: Line-search may reduce step-sizes, if integration failure is encountered, but will finally accept one of the proposed steps after (at least in our study) three iterations. If the ODE can yet not be integrated, the rescue interceptor will be triggered in the next optimization step. It also occurs that the ODE can be integrated for a given parameter vector, but the numerical computation of its gradient is not possible. Also such a case will trigger the rescue interceptor, despite line-search being used.

Algorithm 5 Rescue functionality for mini-batch optimization

Code parts specific to rescue functionality are shown in blue

Initialization:

Set initial parameter vector $\theta \leftarrow \theta_0$
Set and initialize optimization algorithm for parameter update
Set stopping criterion, e.g., maximum number of epochs N
Set variables to store necessary information about previous optimization step: parameter vector θ^*, gradient estimate g^*, possibly optimization algorithm dependent quantities Q^*, initialize with $(\theta^*, g^*, Q^*) \leftarrow (\text{NaN}, \text{NaN}, \text{NaN})$
Set reduction factor $\beta = 1$
Set reduction multiplier $r < 1$
Set increase multiplier $c > 1$

Procedure for optimization with rescue functionality:

while Stopping criterion not met **do**
  Get next mini-batch
  Compute gradient estimate: $g \leftarrow \text{gradientFunction}(\theta)$
  if ODE integration was successful **then**
    Update old quantities: $(\theta^*, g^*, Q^*) \leftarrow (\theta, g, Q)$
    Update reduction factor: $\beta \leftarrow \min(c \cdot \beta, 1)$
    Call parameter updater: $\theta \leftarrow \text{parameterUpdater}(\theta, g, Q, \beta)$
  else
    if θ^* is NaN **then**
      return ODE integration failure at initial point
    end if
    while Last call to gradientFunction failed **and** maximum number of rescue steps not reached **do**
      Undo last step: $(\theta, g, Q) \leftarrow (\theta^*, g^*, Q^*)$
      Update reduction factor $\beta \leftarrow r \cdot \beta$
      Call parameter updater: $\theta \leftarrow \text{parameterUpdater}(\theta, g, Q, \beta)$
      Compute gradient estimate: $g \leftarrow \text{gradientFunction}(\theta)$
    end while
    if Last call to gradientFunction failed **then**
      return ODE integration failure not recoverable
    end if
  end if
end while

Changes in parameterUpdater-*functions*:

Replace parameter update $(\theta \leftarrow \theta + \eta \cdot \delta)$ by $(\theta \leftarrow \theta + \eta \cdot \beta \cdot \delta)$

Algorithm 6 Line-search functionality for mini-batch optimization

Code parts specific to line-search functionality are shown in blue

Initialization:

Set initial parameter vector $\theta \leftarrow \theta_0$
Set and initialize optimization algorithm for parameter update
Set stopping criterion, e.g., maximum number of epochs N
Set variable to store information from previous optimization step: parameter vector θ^*

Procedure for optimization with line-search functionality:

while Stopping criterion not met **do**
    Get next mini-batch
    Compute objective and gradient estimate: $\left(j, g\right) \leftarrow \text{gradientFunction}(\theta)$
    Set line-search reduction factor $\gamma = 1$
    Call parameter updater: $\theta \leftarrow \text{parameterUpdater}(\theta, g, Q, \gamma)$
    Compute objective: $j^{(1)} \leftarrow \text{objectiveFunction}(\theta)$
    if Improvement: $j^{(1)} < j$ **then**
        Accept step: $\theta^* \leftarrow \theta$
    else
        Undo last step: $\theta \leftarrow \theta^*$
        Compute optimal line-search factor $\gamma \leftarrow$ based on quadratic interpolation for $g, j, j^{(1)}$
        Call parameter updater: $\theta \leftarrow \text{parameterUpdater}(\theta, g, Q, \gamma)$
        Compute objective: $j^{(2)} \leftarrow \text{objectiveFunction}(\theta)$
        if Improvement: $j^{(2)} < j$ **then**
            Accept step: $\theta^* \leftarrow \theta$
        else
            Undo last step: $\theta \leftarrow \theta^*$
            Compute optimal line-search factor $\gamma \leftarrow$ based on cubic interpolation for $g, j, j^{(1)}, j^{(2)}$
            Call parameter updater: $\theta \leftarrow \text{parameterUpdater}(\theta, g, Q, \gamma)$
            Compute objective: $j^{(3)} \leftarrow \text{objectiveFunction}(\theta)$
            Set $m = 3$
            while No improvement **and** maximum number of line-search steps not reached **do**
                Undo last step: $\theta \leftarrow \theta^*$
                Compute optimal line-search factor $\gamma \leftarrow$ based on cubic interpolation for $g, j, j^{(m-1)}, j^{(m)}$
                Call parameter updater: $\theta \leftarrow \text{parameterUpdater}(\theta, g, Q, \gamma)$
                Compute objective: $j^{(m+1)} \leftarrow \text{objectiveFunction}(\theta)$
                Increment $m \leftarrow m + 1$
            end while
            Accept step: $\theta^* \leftarrow \theta$
        end if
    end if
end while

Changes in parameterUpdater-*functions*:

Replace parameter update $(\theta \leftarrow \theta + \eta \cdot \delta)$ by $(\theta \leftarrow \theta + \eta \cdot \gamma \cdot \delta)$

4 Proliferation output of the large-scale pan-cancer model

The ODE model of the large-scale application example (3) is mostly built around RAS and AKT singaling. It implements overall 107 genes and s series of specific mutations and the corresponding proteins. Most proteins can be phosphorylated at different phosphorylation sites and some of the phosphorylated downstream components are integrated in the viability output of the model. The corresponding model states enter this output as either beneficial (being denoted as x_i^P, for the i-th such state) or adverse (being denoted as x_i^A, for the i-th such state) for viability:

$$y(\theta) = s \cdot \frac{\sum_{i=1}^{n_P} w_i^P \cdot x_i^P(t^*, \theta, u)}{1 + \sum_{i=1}^{n_A} w_i^A \cdot x_i^A(t^*, \theta, u)} \tag{12}$$

Here, s denotes a scaling parameter, which is used to scale the model output to the training data, n_P and n_A are the numbers of pro- or anti-proliferative species in the model, and t^* denotes a late time point, when the model has reached steady state (typically $t = 10^8$). Every state, which enters the viability output, carries a weight $w_i^[P|A]$, which is inferred from the measurements data, ie., optimized along with the other model parameters. The states entering as the readout in a pro-viability sense are:

- double phosphorylated (S324, S383) ELK1 (in nucleus)
- triple phosphorylated FOS (S374, T325, T331) - double phosphorylated JUN (S63, S73) complex (in nucleus)
- double phosphorylated JUN (S63, S73) homo-dimer (in nucleus)
- phosphorylated STAT5B (Y699) homo-dimer (in nucleus)
- double phosphorylated ATF2 (T69, T71) - double phosphorylated JUN (S63, S73) complex (in nucleus)
- double phosphorylated FOSL1 (S252, S265) - double phosphorylated JUN (S63, S73) complex (in nucleus)
- phosphorylated STAT3 (Y705) homo-dimer (in nucleus)
- phosphorylated CREB1 (S133) (in nucleus)
- phosphorylated MYC (S62) - MAX complex (in nucleus)
- phosphorylated STAT1 (Y701) homo-dimer (in nucleus)
- phosphorylated STAT5A (Y694) homo-dimer (in nucleus)
- phosphorylated MYCN (S62) - MAX complex (in nucleus)

and the states which enter as anti-proliferative are:

- FOXO1 protein (in nucleus)
- FOXO3 protein (in nucleus)
- FOXO4 protein (in nucleus)
- FOXO6 protein (in nucleus)

5 Interpretation and discussion of the in-silico knockout study

Despite being large in scale, the cancer signaling model from the application example is still a simplification of reality. In particular, some of its downstream components are implemented for completeness, but actually constitute ”dead-ends” that do not affect the model output. Especially such genes which are implemented as such ”dead-ends” were often incorrectly predicted as being non-essential in the in-silico knockout study. Hence, based on the results from the in-silico knockouts with the original model, the viability output was refined. In this refined version, the following two species were added to the list of pro-proliferative model states:

- CHEK1 protein (in cytoplasm)
- double phosphorylated MAP3K1 (T1400, T1412) (in cytoplasm)

This refined model was calibrated to the original drug response data (13,000 data points) and then used for in-silico knockout predictions. These knockout predictions, as well as the number of true/false positive/negative predictions, are shown in Supplementary Fig. 12, for the 18 considered cell lines. The results of these in-silico knockouts were clearly superior to the ones of the original model (Supplementary Fig. 11). Yet, a series of major false positive and false negative predictions persisted, which we want to discuss in the following. We thereby restrict to genes, which had incorrect predictions for at least 20% of the considered cell lines (i.e., at least 4 out of 18 cell lines).

False positive predictions

Most false positive predictions can be explained by the fact that the model is, despite being large, still a strong simplification of reality. The computational model implements a number of distinct pathways, which transduce a signal from the cell membrane to the nucleus, resulting in a potentially changed cell viability. However, a real cell has more possibilities to react to perturbations than any computational model. Hence, the importance of the proteins, which play key roles in signal transduction within the model (in this case in AKT and RAS signaling related species), will typically be overestimated. Most of the false positive predictions of the trained model can be attributed to this effect. A particular group of such false positive predictions concerns those genes, which encode for proteins that bind to EGF receptor complexes. Important false positive predictions were made for

- STAT3 (18 out of 18 cell lines)
- SRC (18 out of 18 cell lines)
- RAF1 (18 out of 18 cell lines)
- MAPK3 (18 out of 18 cell lines)
- MAPK1 (18 out of 18 cell lines)
- MAP2K1 (18 out of 18 cell lines)
- ELK1 (18 out of 18 cell lines)
- EGFR (18 out of 18 cell lines)
- CREB1 (18 out of 18 cell lines)
- NRAS (17 out of 18 cell lines)

- BRAF (17 out of 18 cell lines)
- MAP2K2 (17 out of 18 cell lines)
- VAV2 (16 out of 18 cell lines)
- STAT5B (16 out of 18 cell lines)
- JUN (15 out of 18 cell lines)
- SOS1 (14 out of 18 cell lines)
- PAK1 (14 out of 18 cell lines)
- ADAM17 (13 out of 18 cell lines)
- MAP3K1 (13 out of 18 cell lines)
- MAX (12 out of 18 cell lines)
- ERBB2 (11 out of 18 cell lines)
- STAT1 (9 out of 18 cell lines)
- ERBB4 (8 out of 18 cell lines)
- ADAM10 (8 out of 18 cell lines)
- STAT5A (7 out of 18 cell lines)
- SHC1 (7 out of 18 cell lines)
- EGF (6 out of 18 cell lines)
- GRB2 (6 out of 18 cell lines)
- RAC1 (6 out of 18 cell lines)
- ATF2 (6 out of 18 cell lines)
- AREG (5 out of 18 cell lines)
- TGF-α (4 out of 18 cell lines)
- PIK3R1 (4 out of 18 cell lines)
- GAB1 (4 out of 18 cell lines)
- BTC (4 out of 18 cell lines)

EGF receptor binding: EGFR, ERBB2, TGF-α, SRC, SHC1, VAV2, SOS1, GRB2, GAB1, PIK3RC The proteins of these genes are implemented as being active in the formation and the signaling at the receptor level, in particular of the EGF and the AREG receptor complexes. Although the present model incorporates multiple receptor complexes, such as homo-dimers of EGFR, ERBB2, and ERBB4, and heterodimers of ERBB2 with EGFR, ERBB3, and ERBB4, the EGF receptor complexes were estimated to be most important for cell viability during model calibration. Interestingly, proteins such as VAV2, SOS1, GRB, and GAB1 also form complexes with ERBB3 and ERBB4, but so do the proteins of NRG1, NRG2, NRG3, and NRG4, which were (correctly) not estimated to be as essential by the model. Hence, we assume that the false positive predictions for the in-silico knockouts of VAV2, SOS1, GRB, and GAB1 are due to their role in the signal transduction downstream from EGFR. Another indication, that these false positives stem from overestimating the essentiality of EGFR is the prominent appearance of SRC (FP for 18 out of 18 cell lines), which binds almost exclusively to EGFR complexes in the model.

The reason for this strong bias for EGFR-related genes may either be due to the model topology, or due to the dataset on which the model was trained. Since the model also implements many other receptor complexes, which carry out similar functionality, it is likely that not only the model topology is biased, but also the dataset which was used for training. This sounds plausible, as many of the implemented drugs target the EGF receptor complexes and hence, model calibration will be biased towards overestimating their importance, as they are probably over-represented in the training dataset.

Cleavage proteins: ADAM17 and ADAM10 The two proteins of ADAM10 and ADAM17 cleave the receptor complexes from the ligands. In a real cell, this functions most likely also carried out by other proteins, although ADAM10 and ADAM17 are probably the most prominent ones. Indeed, knockout out ADAM10 or ADAM17 led to reduced cell viability in 10 and 6 cell lines out of 18, respectively. However, the present model predicts these reductions to be more pronounced than they are in the experimental data. On one hand, it may be that these two proteins are over-represented in the model, as they are the only ones which are responsable for receptor cleavage. On the other hand, we assume that the general bias of the trained models towards the EGFR complexes also causes those two cleavage proteins to be overestimated in essentiality.

ERBB4 Beyond receptors that form dimers with EGFR, also ERBB4 was estimated to be essential in 8 out of 18 cell lines. In the experimental data, a knockout of ERBB4 led indeed to reduced cell viability in 12 out of 18 cell lines with an average viability reduction of 12%. However, this was not enough to be classified as an essential reduction. Hence, the model is still overestimates the essentiality of ERBB4, probably for similar reasons as in the case of the EGFR, although this effect is less pronounced for ERBB4.

Growth factors: EGF, AREG, BTC The proteins, for which these genes encode, are growth factors that bind among others to the EGF receptor complexes. Hence, again due to the general over-representation of EGFR-related genes, also EGF, AREG, and BTC are found among the false positive predictions of the model.

RAS/RAF/MEK/ERK cascade: NRAS, PAK1, RAC1, MAP3K1, RAF1, BRAF, MAP2K1, MAP2K2, MAPK1, MAPK3 The main signaling pathway transducing the growth signal downwards is RAS/RAF/MEK/ERK cascade. While any perturbation on its main proteins does indeed reduce cell viability also in experimental data, this reduction is more pronounced in the present model than in the experiment. Again, we assume this to be due the fact that this model is still a strong simplification of the actual processes in a cancer cell. Typically, a living cell still has more possibilities to circumvent perturbations in this signaling cascade than the present model allows for. Hence, the essentiality of these proteins is generally over-estimated,

which is not surprising. However, RAC1 has a particular role in this list of proteins, as the model implements it as being very similar to CDC42, which was however under-estimated in essenitality. Hence, we can assume that the data, on which the model was trained, did not allow to clearly discern the roles of these two proteins during model calibration.

Transcription factors (single proteins and homo-dimers): ELK1, CREB1, STAT3, STAT5B, STAT1, STAT5A These genes encode for transcription factors, which are implemented as enhancing proliferation in the model. Hence, a knockout of those will reduce the prediction of cell viability, which makes them particularly prone to being candidates of false positive predictions. For ELK1, experimental data showed indeed an average reduction of cell viability of 20% across the 18 cell lines, while these effects were less pronounced for CRAB1 and the STAT family. A real cell, in which one of these genes is knocked out, would compensate for the effect of the knockout to a certain degree, such that a knockout does not need to cause a reduction of cell viability. However, such compensating mechanism are not possible in such a computational model.

Transcription factors (as protein complexes): JUN, MAX, ATF2 In the present model, JUN contributes in form of complexes, e.g., with ATF2, to an increased cell viability. Hence, the model considers it to be curcial for cell viability. In 10 out of the 18 cell lines, a JUN knockout leads indeed to decreased cell viability of 17% in average. For MAX, the situation is different, as it contributes to cell viability in form of the MYC:MAX complex. Hence, the model captures cellular behavior, but overestimates the importance of the implemented genes.

False negative predictions

False negative predictions give hints, were the model may neglect aspects that are biologically relevant. Therefore, they are particularly interesting for model refinements. Important false negative predictions were made for

- CDC42 (9 out of 18 cell lines)
- PDPK1 (8 out of 18 cell lines)
- PPP2CA (7 out of 18 cell lines)
- WNK1 (4 out of 18 cell lines)

CDC42 The protein of CDC42 is, similar to RAC1, a GTPase. Both species carry out similar function in the model. Interestingly, the effect of knocking out RAC1 was overestimated, while the effect of knocking out CDC42 was underestimated. Hence, it seems likely that the similar role of both proteins did not allow to reliably distinguish between their function during model training and hence, the model "confuses" both genes to a certain degree.

PDPK1 The protein PDPK1 is a master kinase, which is actively involved in many reactions in the cell, expecially in cancer signaling. In the present model, PDPK1 phosphorylates the proteins AKT1/2/3, which influence the proliferation. As there are also other ways to achieve this phosphorylation in the model, PDPK1 is estimated to be essential only for few cell lines. However, in a real cell, PDPK1 regulates substantially processes, which are not reflected in the model. This may explain why the effect of a PDPK1 knockout on cell viability is underestimated.

PPP2CA PPP2CA is a phosphatase which is active in various processes. However, in the present model, it dephosphorylates AKT1 and MAP2K1. Hence, only a small fraction of the processes, in which PPP2CA is involved, is captured compared to reality. Hence, its importance is overall underestimated.

WNK1 The protein WNK1 regulates cation cotransporters, which mediate, among others, homeostasis. Hence, a WNK1 knockout will be fatal for many cell lines. However, as the current model is concerned with signal transduction and not ionic transport, it does not reflect the functioning of WNK1, but just implements it as a "dead-end" in the downstream target of AKT. Hence, WNK1 can not be captured as an essential gene by this model.

6 Possible proliferation models and refinements for the viability readout

The in-silico knockout study has shown that capturing cell cycle is crucial to correctly predict the outcome of gene knockouts. As cell cycle, viability, and proliferation are tightly linked, it makes sense to extend the current signaling model by a proliferation model, which is more complex than the viability readout given in (12). Although a detailed analysis of possible proliferation models is beyond the scope of this work, we want to briefly discuss some extension and their possible implementations, which might be a starting point for future work.

General Framework

The main idea of the subsequent proliferation models is to describe proliferation via cell counts, which are part of the model and not just as one of its observables. This means defining cell counts as an own species N, which takes part in two reactions: cell division and cell death. Building upon the idea of pro- and anti-proliferative species we would divide signalling species with an effect on proliferation/apoptosis into the four groups P_+, P_-, A_+, A_- corresponding to pro-/anti-proliferative species and pro-/anti-apoptotic species respectively. With reaction rate constants α, β depending on these sets of species we obtain the system

$$\text{Cell Division: } N \xrightarrow{\alpha(P_\pm)} 2N$$
$$\text{Cell Death: } N \xrightarrow{\beta(A_\pm)} \emptyset.$$

Initial cell counts $N(0)$ can either be obtained directly from measurements or estimated as parameters from later time points. Following the idea of (12) the reaction rate constants could be defined as follows

$$\alpha(P_\pm) = \frac{\sum_{s \in P_+} w_s * [s]}{1 + \sum_{s \in P_-} w_s * [s]} \qquad \beta(A_\pm) = \frac{\sum_{s \in A_+} w_s * [s]}{1 + \sum_{s \in A_-} w_s * [s]}$$

for some species dependent weights w_s. These weight would be estimated along with the remaining model parameters, similar to the current implementation of the viability readout. In the next paragraphs we will discuss two ways of defining the reaction rates and some adjustments to the setup above.

Growth models

Exponential growth model The exponential growth model assumes that the measurements are in a time regime where cells can proliferate unboundedly and have not reached a saturation level yet. However, this

makes this growth model incompatible with, e.g., viability data taken at steady-state. With the reaction rate constants defined as above we obtain the exponential growth ODE:

$$\frac{d}{dt}N(t) = \frac{\sum_{s\in P_+} w_s * [s]}{1+\sum_{s\in P_-} w_s * [s]}N(t) - \frac{\sum_{s\in A_+} w_s * [s]}{1+\sum_{s\in A_-} w_s * [s]}N(t)$$

Logistic growth model The logistic growth model tackles the assumption of unlimited growth by introducing a saturation level at a maximum cell number N_{max}. The ODE for the logistic growth reads:

$$\frac{d}{dt}N(t) = \frac{\sum_{s\in P_+} w_s * [s]}{1+\sum_{s\in P_-} w_s * [s]}(N(t) - N(t)^2/N_{max}) - \frac{\sum_{s\in A_+} w_s * [s]}{1+\sum_{s\in A_-} w_s * [s]}N(t),$$

A downside of this approach could be that for some initial parameters the calibration starts at the flat parts of the logistic curve, where close to zero gradients would prevent improvement of parameters.

Possible further improvements

Saturation of Species Effects So far we have assumed that the effect a high concentration of a species can have on proliferation/apoptosis is unlimited. However, in reality it might be more realistic to assume that this effect saturates at some maximum effect level. Assuming a Michaelis-Menten (MM) like saturation of the species effects on proliferation would yield for example a logistic growth ODE

$$\frac{d}{dt}N(t) = \frac{\sum_{s\in P_+} v_s * \frac{[s]}{k_s+[s]}}{1+\sum_{s\in P_-} v_s * \frac{[s]}{k_s+[s]}}(N(t) - N(t)^2/N_{max}) - \frac{\sum_{s\in A_+} w_s * [s]}{1+\sum_{s\in A_-} w_s * [s]}N(t),$$

with some parameters k_s, v_s.

Cell Count Transformations Depending on initial parameters, exponential growth, but to some extent also logistic growth, can grow rapidly and cause numerical problems. Hence log-transformations of the cell count species and measurements could prove beneficial. Defining $\tilde{N} = \log N$ yields for, e.g., the exponential growth model:

$$\frac{d}{dt}\tilde{N} = \frac{\frac{d}{dt}N}{N} = \frac{1}{N}(\alpha N - \beta N) = (\alpha - \beta)$$

Growth models could be investigated by performing a study, which investigates, e.g., the following combinations:

$$\begin{pmatrix}\text{exp. growth}\\ \text{log. growth}\end{pmatrix} \times \begin{pmatrix}\text{with MM sat.}\\ \text{without sat.}\end{pmatrix} \times \begin{pmatrix}\text{log cell count}\\ \text{linear cell count}\end{pmatrix}$$

Supplementary References

[1] F. M. Behan, F. Iorio, G. Picco, E. Gonçalves, C. M. Beaver, G. Migliardi, R. Santos, Y. Rao, F. Sassi, M. Pinnelli Prioritization of cancer therapeutic targets using CRISPR–Cas9 screens. *Nature*, 568(7753), 2019.

[2] F. Fröhlich, B. Kaltenbacher, F. J. Theis, and J. Hasenauer. Scalable parameter estimation for genome-scale biochemical reaction networks. *PLoS Comput. Biol.*, 13(1):e1005331, 2017.

[3] F. Fröhlich, T. Kessler, D. Weindl, A. Shadrin, L. Schmiester, H. Hache, A. Muradyan, M. Schütte, J.-H. Lim, M. Heinig, F. J. Theis, H. Lehrach, C. Wierling, B. Lange, and J. Hasenauer. Efficient parameter estimation enables the prediction of drug response using a mechanistic pan-cancer pathway model. *Cell Syst.*, 7(6):567–579.e6, 2018.

[4] I. Goodfellow, Y. Bengio, and A. Courville. *Deep Learning*. MIT Press, 2016.

[5] D. Kaschek, W. Mader, M. Fehling-Kaschek, M. Rosenblatt, and J. Timmer. Dynamic modeling, parameter estimation, and uncertainty analysis in r. *J. Stat. Softw.*, 88(10), 2019.

[6] D. P. Kingma and L. J. Ba. Adam: A method for stochastic optimization. In *International Conference on Learning Representations (ICLR) 2015 - accepted papers*. Ithaca, 2015.

[7] E. Klipp, R. Herwig, A. Kowald, C. Wierling, and H. Lehrach. *Systems biology in practice*. Wiley-VCH, Weinheim, 2005.

[8] P. Mendes, S. Hoops, S. Sahle, R. Gauges, J. Dada, and U. Kummer. *Computational Modeling of Biochemical Networks Using COPASI*, chapter 2. Part of the Methods in Molecular Biology. Humana Press, 2009.

[9] J. Nocedal and S. Wright. *Numerical Optimization*. Springer Science & Business Media, 2006.

[10] B. T. Polyak. Some methods of speeding up the convergence of iteration methods. *USSR Comp. Math. Math. Phys.*, 4(5):1–17, 1964.

[11] A. Raue, M. Schilling, J. Bachmann, A. Matteson, M. Schelke, D. Kaschek, S. Hug, C. Kreutz, B. D. Harms, F. J. Theis, U. Klingmüller, and J. Timmer. Lessons learned from quantitative dynamical modeling in systems biology. *PLoS ONE*, 8(9):e74335, 2013.

[12] A. Raue, B. Steiert, M. Schelker, C. Kreutz, T. Maiwald, H. Hass, J. Vanlier, C. Tönsing, L. Adlung, R. Engesser, W. Mader, T. Heinemann, J. Hasenauer, M. Schilling, T. Höfer, E. Klipp, F. J. Theis, U. Klingmüller, B. Schöberl, and J.Timmer. Data2Dynamics: a modeling environment tailored to parameter estimation in dynamical systems. *Bioinformatics*, 31(21):3558–3560, 2015.

[13] H. Robbins and S. Monroe. A stoachstic approximation method. *Ann. Math. Stat.*, 22(3):400–407, 1951.

[14] S. Ruder. An overview of gradient descent optimisation algorithms. arXiv:1609.04747.

[15] Y. Schälte, P. Stapor, and J. Hasenauer. Evaluation of derivative-free optimizers for parameter estimation in systems biology. *IFAC-PapersOnLine*, 51(19):98–101, 2018.

[16] L. Schmiester, Y. Schälte, F. Fröhlich, J. Hasenauer, and D. Weindl. Efficient parameterization of large-scale dynamic models based on relative measurements. *Bioinformatics*, btz581, 07 2019.

[17] I. Sutskever, J. Martens, G. Dahl, and G. Hinton. On the importance of initialization and momentum in deep learning. In *Proc. Int. Conf. Machine Learning*, pages 1139–1147, 2013.

[18] L. Tieleman and G. Hinton. Lecture 6.5 – rmsprop: Divide the gradient by a running average of its recent magnitude. COURSERA: Neural Networks for Machine Learning, 2012.

[19] T. McInnes, J. Healy, N. Saul, and L. Grossberger UMAP: Uniform Manifold Approximation and Projection. *The Journal of Open Source Software*, 3(29):861, 2018.

[20] A. F. Villaverde, F. Froehlich, D. Weindl, J. Hasenauer, and J. R. Banga. Benchmarking optimization methods for parameter estimation in large kinetic models. *Bioinformatics*, page bty736, 2018.

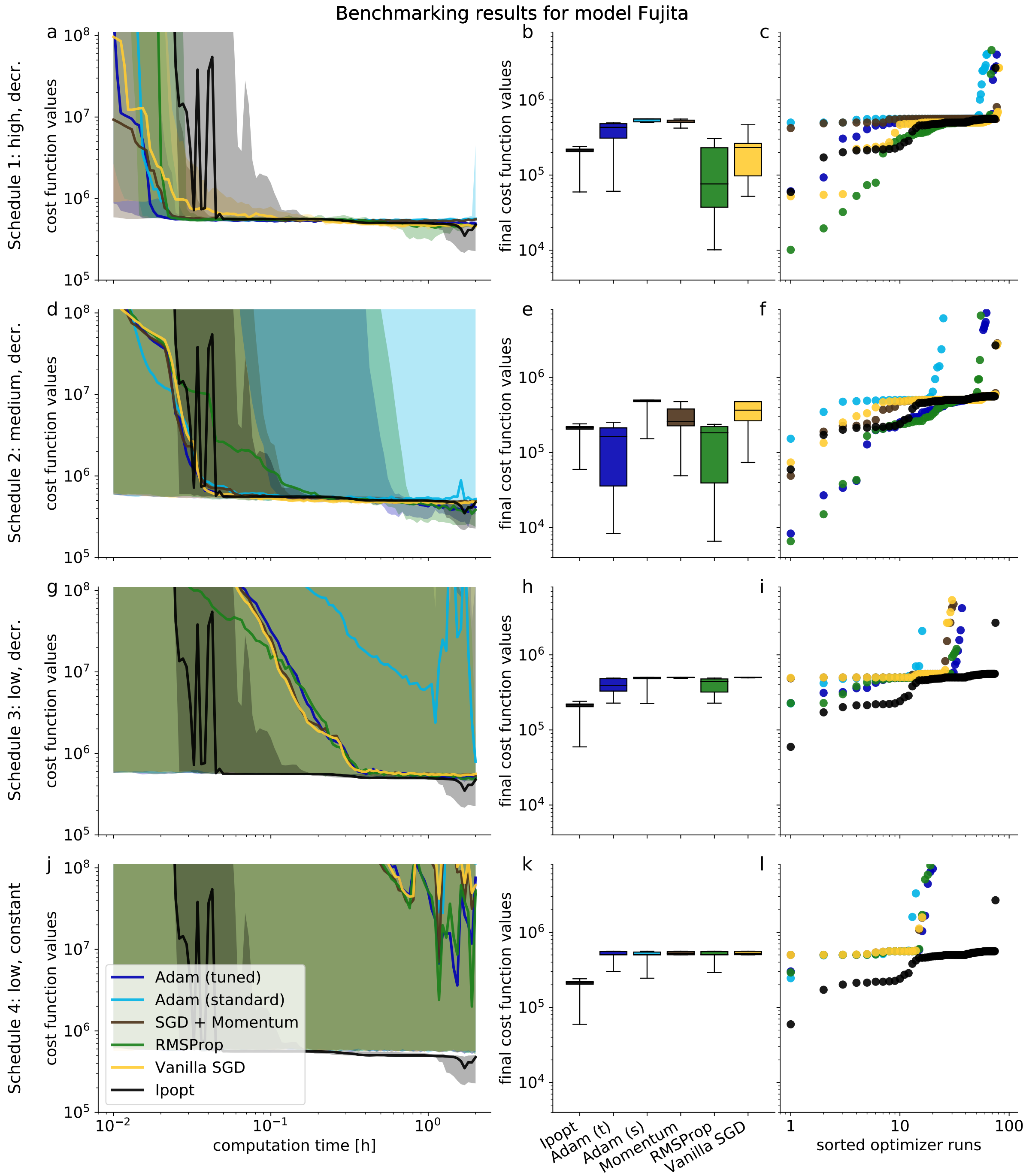

Supplementary Figure 1: Optimization results for different learning rate Schedules, benchmarked against the full-batch optimizer Ipopt on Fujita example model (Supplementary to Fig. 2 of main manuscript). Objective function values along optimization traces were extrapolated from mini-batch to the full dataset, smoothed over multiple optimization steps to filter the stochasticity of the mini-batches, and interpolated for a mesh of computation time points. Shading depicts percentiles for 10% and 40% of optimizer traces, solid lines depict percentiles for 20%. Final objective function values for box and waterfall plots were evaluated at final point of optimization trajectory. **a** Traces of objective function value during optimization for learning rate Schedule 1. **b** Box plots of the 10 best final objective function values learning rate Schedule 1. **c** Waterfall plot of all final objective function values for learning rate Schedule 1. **d - f** Analogue to subfigures (a - c), for learning rate Schedule 2. **g - i** Analogue to subfigures (a - c), for learning rate Schedule 3. **j - l** Analogue to subfigures (a - c), for learning rate Schedule 4.

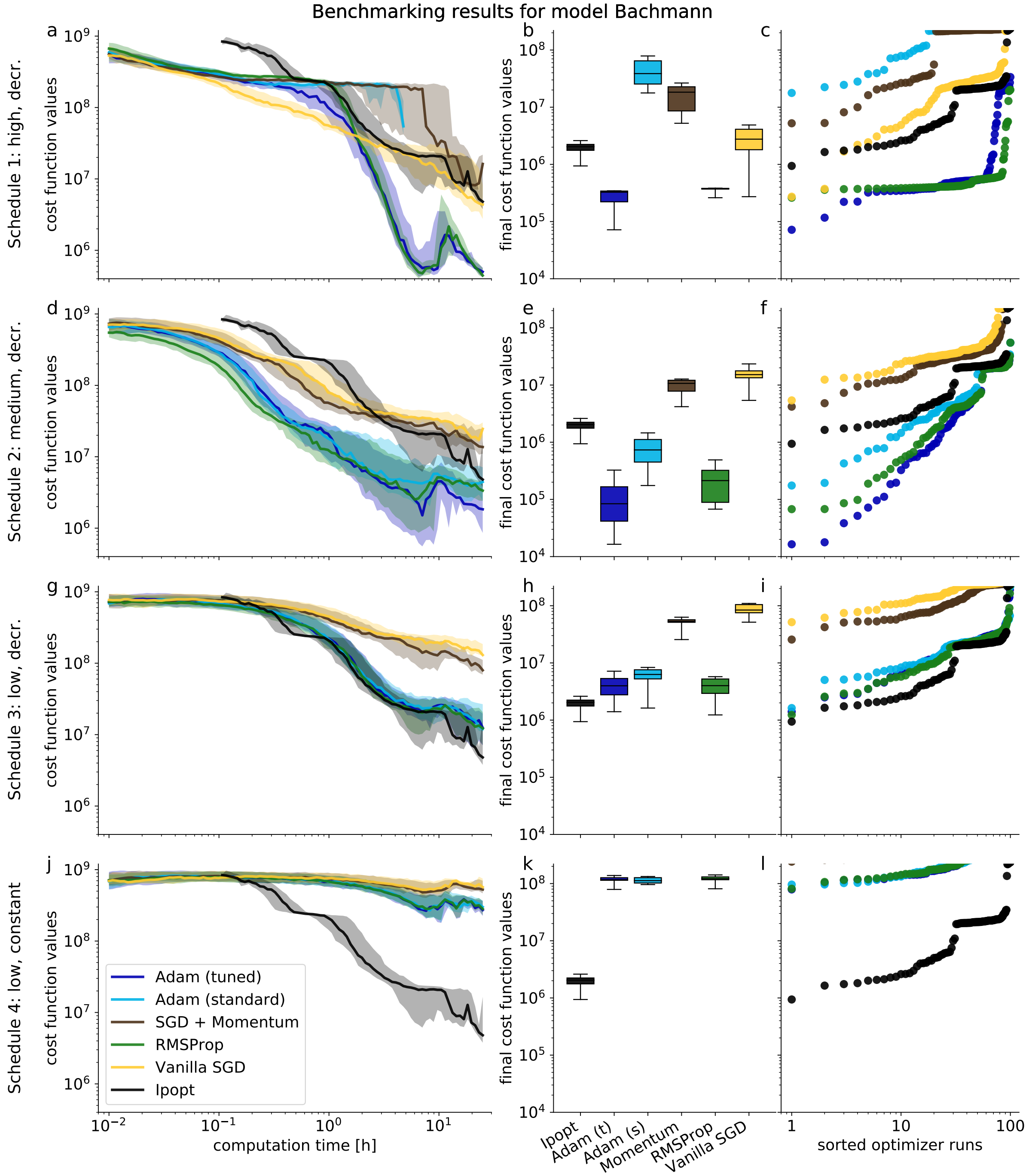

Supplementary Figure 2: Optimization results for different learning rate Schedules, benchmarked against the full-batch optimizer Ipopt on Bachmann example model (Supplementary to Fig. 2 of main manuscript). Objective function values along optimization traces were extrapolated from mini-batch to the full dataset, smoothed over multiple optimization steps to filter the stochasticity of the mini-batches, and interpolated for a mesh of computation time points. Shading depicts percentiles for 10% and 40% of optimizer traces, solid lines depict percentiles for 20%. Final objective function values for box and waterfall plots were evaluated at final point of optimization trajectory. **a** Traces of objective function value during optimization for learning rate Schedule 1. **b** Box plots of the 10 best final objective function values learning rate Schedule 1. **c** Waterfall plot of all final objective function values for learning rate Schedule 1. **d - f** Analogue to subfigures (a - c), for learning rate Schedule 2. **g - i** Analogue to subfigures (a - c), for learning rate Schedule 3. **j - l** Analogue to subfigures (a - c), for learning rate Schedule 4.

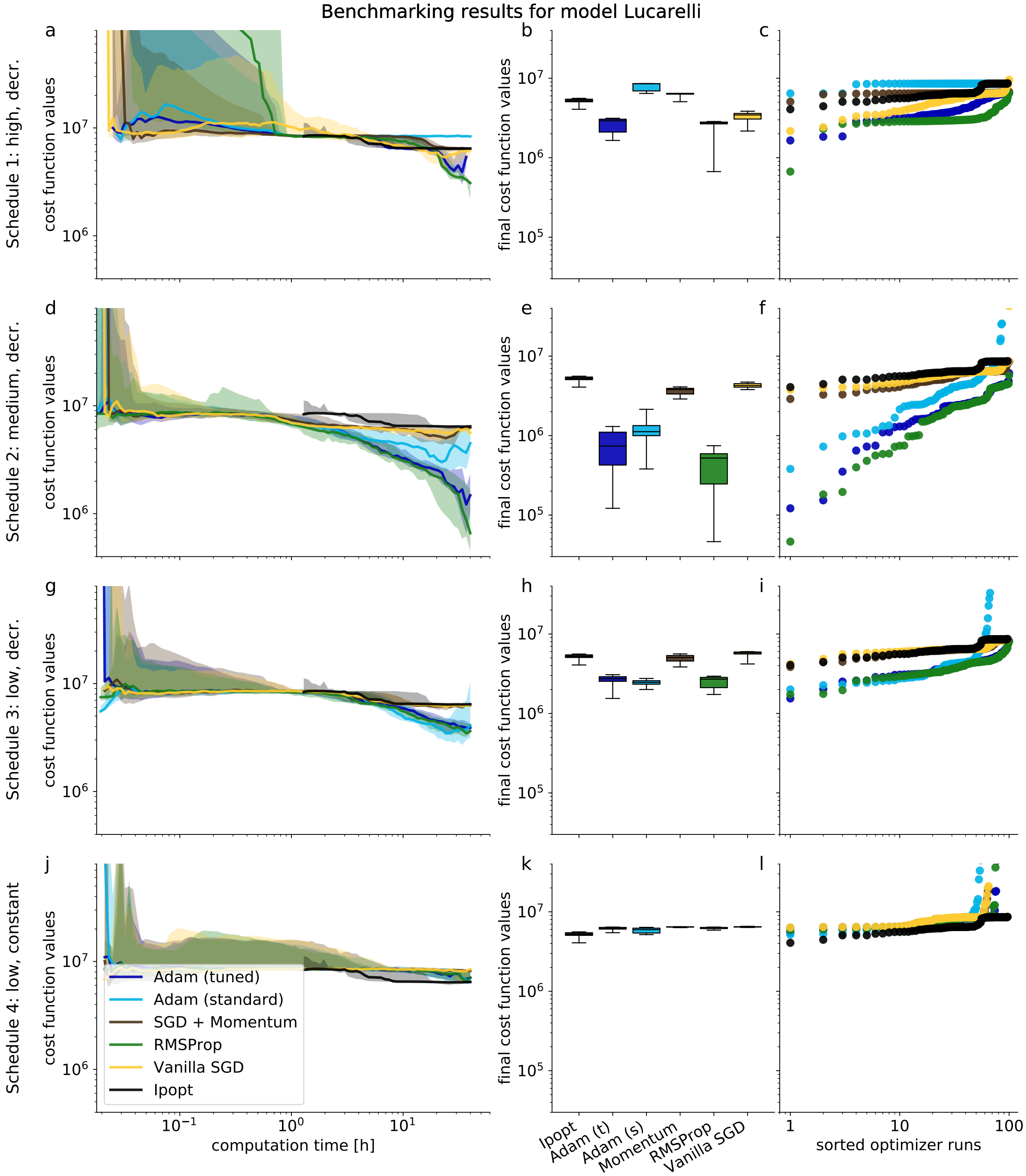

Supplementary Figure 3: Optimization results for different learning rate Schedules, benchmarked against the full-batch optimizer Ipopt on Lucarelli example model (Supplementary to Fig. 2 of main manuscript). Objective function values along optimization traces were extrapolated from mini-batch to the full dataset, smoothed over multiple optimization steps to filter the stochasticity of the mini-batches, and interpolated for a mesh of computation time points. Shading depicts percentiles for 10% and 40% of optimizer traces, solid lines depict percentiles for 20%. Final objective function values for box and waterfall plots were evaluated at final point of optimization trajectory. **a** Traces of objective function value during optimization for learning rate Schedule 1. **b** Box plots of the 10 best final objective function values learning rate Schedule 1. **c** Waterfall plot of all final objective function values for learning rate Schedule 1. **d - f** Analogue to subfigures (a - c), for learning rate Schedule 2. **g - i** Analogue to subfigures (a - c), for learning rate Schedule 3. **j - l** Analogue to subfigures (a - c), for learning rate Schedule 4.

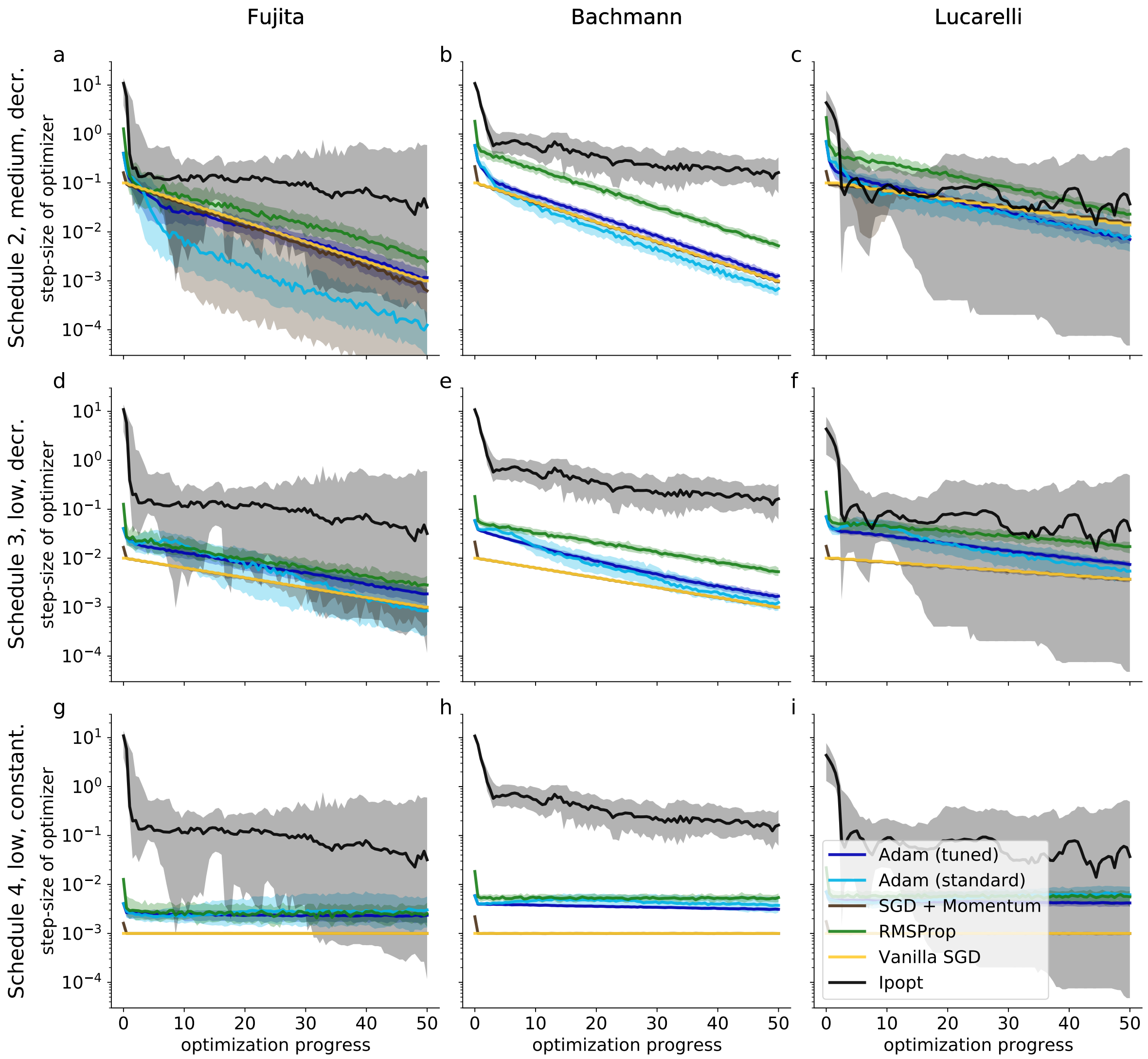

Supplementary Figure 4: Learning rates leading to step-sizes slightly smaller than those of full-batch optimization methods perform best (Supplementary to Fig. 2 of main manuscript). Effective step-sizes of optimization steps in parameter space are plotted for full-batch optimizer Ipopt and different mini-batch optimization algorithms, against optimization progress, normalized to 50 iterations/epochs. Overall, medium, decreasing step-sizes performed best in optimization, showing step-sizes similar to the full-batch optimizer Ipopt. Constant learning rates lead to almost constant step-sizes, even for mini-batch algorithms, which are termed adaptive in literature, i.e., RMSProp and Adam. Shading depict percentiles for 20% and 80% of optimizer traces, solid lines depict medians. **a** Step-sizes for Fujita model for learning rate Schedule 2 (medium, decreasing learning rate). **b** Step-sizes for Bachmann model for learning rate Schedule 2. **c** Step-sizes for Lucarelli model for learning rate Schedule 2. **d** Step-sizes for Fujita model for learning rate Schedule 3. **e** Step-sizes for Bachmann model for learning rate Schedule 3. **f** Step-sizes for Lucarelli model for learning rate Schedule 3. **g** Step-sizes for Fujita model for learning rate Schedule 4. **h** Step-sizes for Bachmann model for learning rate Schedule 4. **i** Step-sizes for Lucarelli model for learning rate Schedule 4.

Benchmarking results for model Fujita

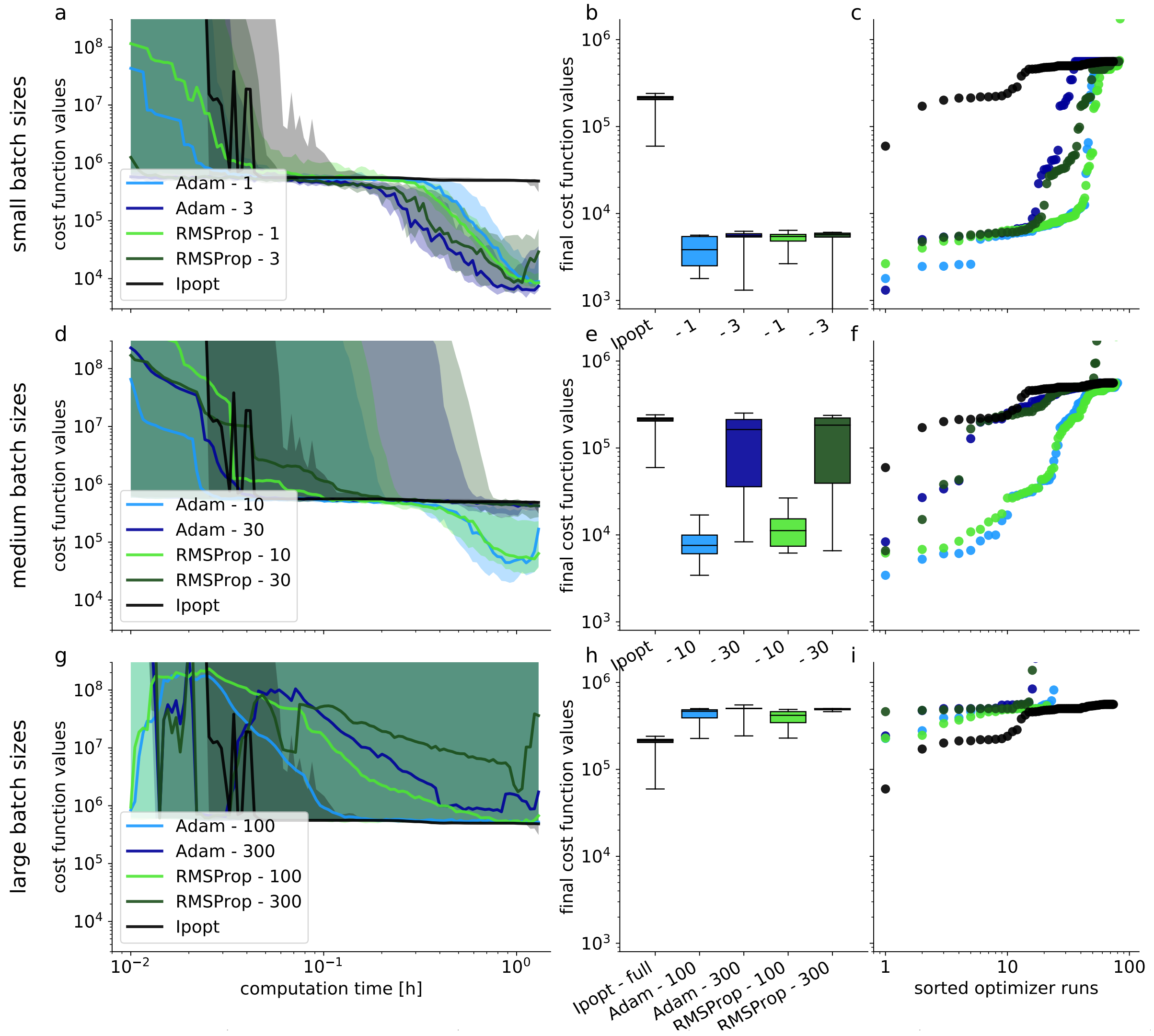

Supplementary Figure 5: Small mini-batch sizes show best performance for mini-batch optimization on Fujita example model (Supplementary to Fig. 2 of main manuscript). Mini-batch optimizers using different mini-batch sizes were benchmarked against full-batch optimizer Ipopt on Fujita example model. Objective function values along optimization traces were extrapolated from mini-batch to the full dataset, smoothed over multiple optimization steps to filter the stochasticity of the mini-batches, and interpolated for a mesh of computation time points. Shading depicts percentiles for 10% and 40% of optimizer traces, solid lines depict percentiles for 20%. Final objective function values for box and waterfall plots were evaluated at final point of optimization trajectory. **a** Traces of objective function value during optimization for small mini-batch sizes. **b** Box plots of the 10 best final objective function values for small mini-batch sizes. **c** Waterfall plot of all final objective function values for small mini-batch sizes. **d** Traces of objective function value during optimization for medium mini-batch sizes. **e** Box plots of the 10 best final objective function values for medium mini-batch sizes. **f** Waterfall plot of all final objective function values for medium mini-batch sizes. **g** Traces of objective function values during optimization with large mini-batch sizes. **h** Box plots of the 10 best final objective function values with large mini-batch sizes. **i** Waterfall plot of all final objective function values with large mini-batch sizes.

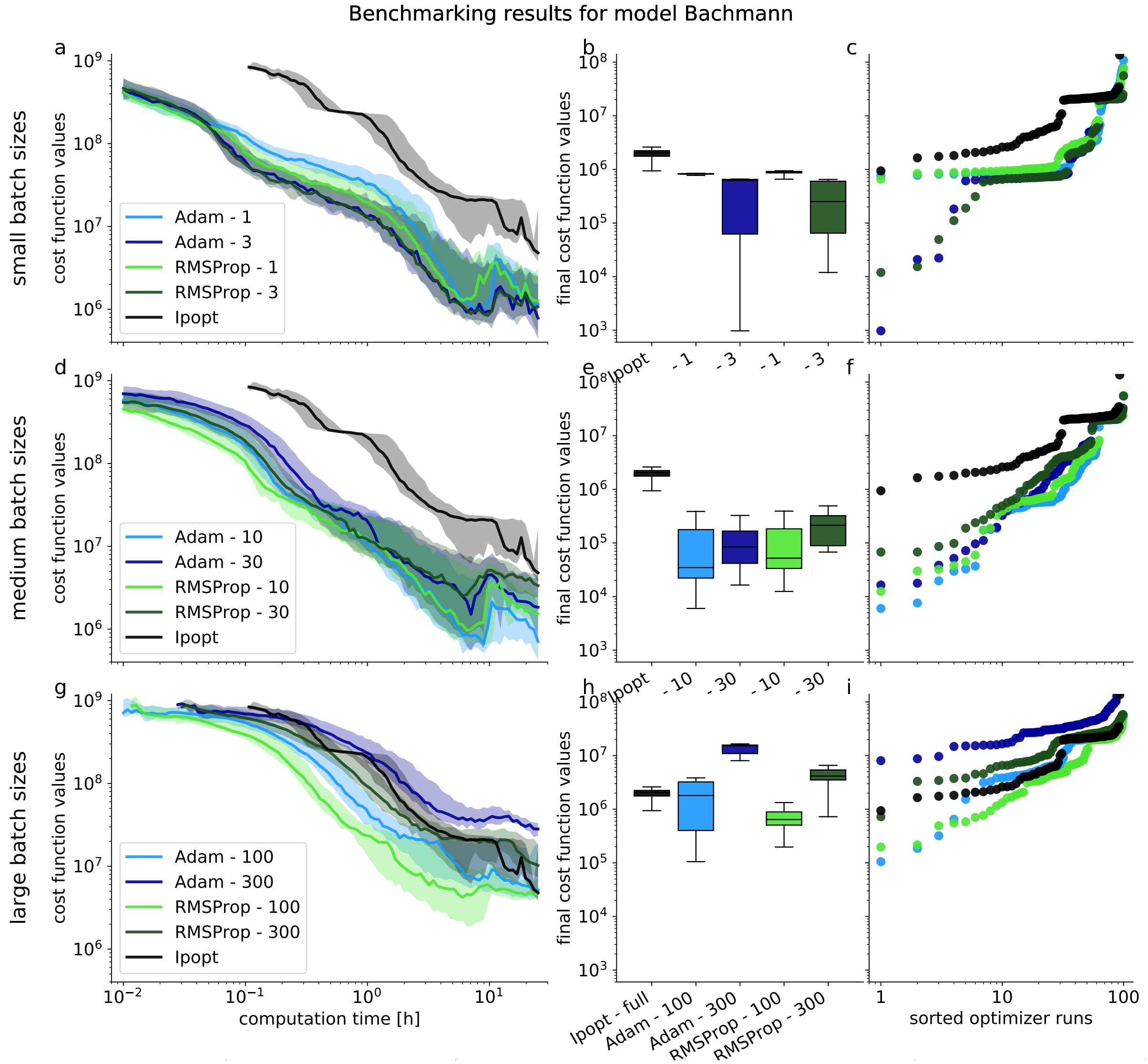

Supplementary Figure 6: Small mini-batch sizes show best performance for mini-batch optimization on Bachmann example model (Supplementary to Fig. 2 of main manuscript). Mini-batch optimizers using different mini-batch sizes were benchmarked against full-batch optimizer Ipopt on Bachmann example model. Objective function values along optimization traces were extrapolated from mini-batch to the full dataset, smoothed over multiple optimization steps to filter the stochasticity of the mini-batches, and interpolated for a mesh of computation time points. Shading depicts percentiles for 10% and 40% of optimizer traces, solid lines depict percentiles for 20%. Final objective function values for box and waterfall plots were evaluated at final point of optimization trajectory. **a** Traces of objective function value during optimization for small mini-batch sizes. **b** Box plots of the 10 best final objective function values for small mini-batch sizes. **c** Waterfall plot of all final objective function values for small mini-batch sizes. **d** Traces of objective function value during optimization for medium mini-batch sizes. **e** Box plots of the 10 best final objective function values for medium mini-batch sizes. **f** Waterfall plot of all final objective function values for medium mini-batch sizes. **g** Traces of objective function values during optimization with large mini-batch sizes. **h** Box plots of the 10 best final objective function values with large mini-batch sizes. **i** Waterfall plot of all final objective function values with large mini-batch sizes.

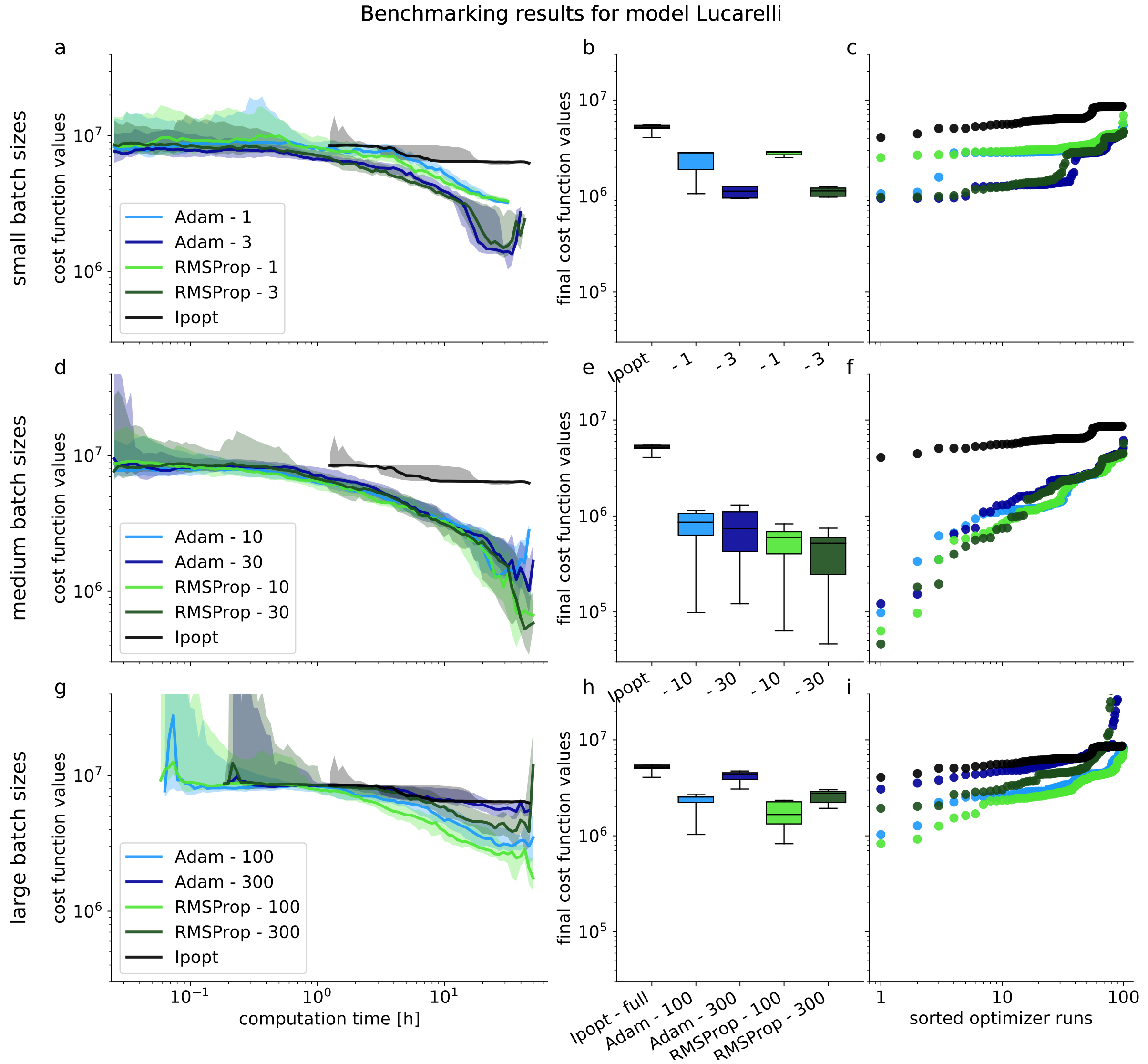

Supplementary Figure 7: Medium mini-batch sizes show best performance for mini-batch optimization on Lucarelli example model (Supplementary to Fig. 2 of main manuscript). Mini-batch optimizers using different mini-batch sizes were benchmarked against full-batch optimizer Ipopt on Lucarelli example model. Objective function values along optimization traces were extrapolated from mini-batch to the full dataset, smoothed over multiple optimization steps to filter the stochasticity of the mini-batches, and interpolated for a mesh of computation time points. Shading depicts percentiles for 10% and 40% of optimizer traces, solid lines depict percentiles for 20%. Final objective function values for box and waterfall plots were evaluated at final point of optimization trajectory. **a** Traces of objective function value during optimization for small mini-batch sizes. **b** Box plots of the 10 best final objective function values for small mini-batch sizes. **c** Waterfall plot of all final objective function values for small mini-batch sizes. **d** Traces of objective function value during optimization for medium mini-batch sizes. **e** Box plots of the 10 best final objective function values for medium mini-batch sizes. **f** Waterfall plot of all final objective function values for medium mini-batch sizes. **g** Traces of objective function values during optimization with large mini-batch sizes. **h** Box plots of the 10 best final objective function values with large mini-batch sizes. **i** Waterfall plot of all final objective function values with large mini-batch sizes.

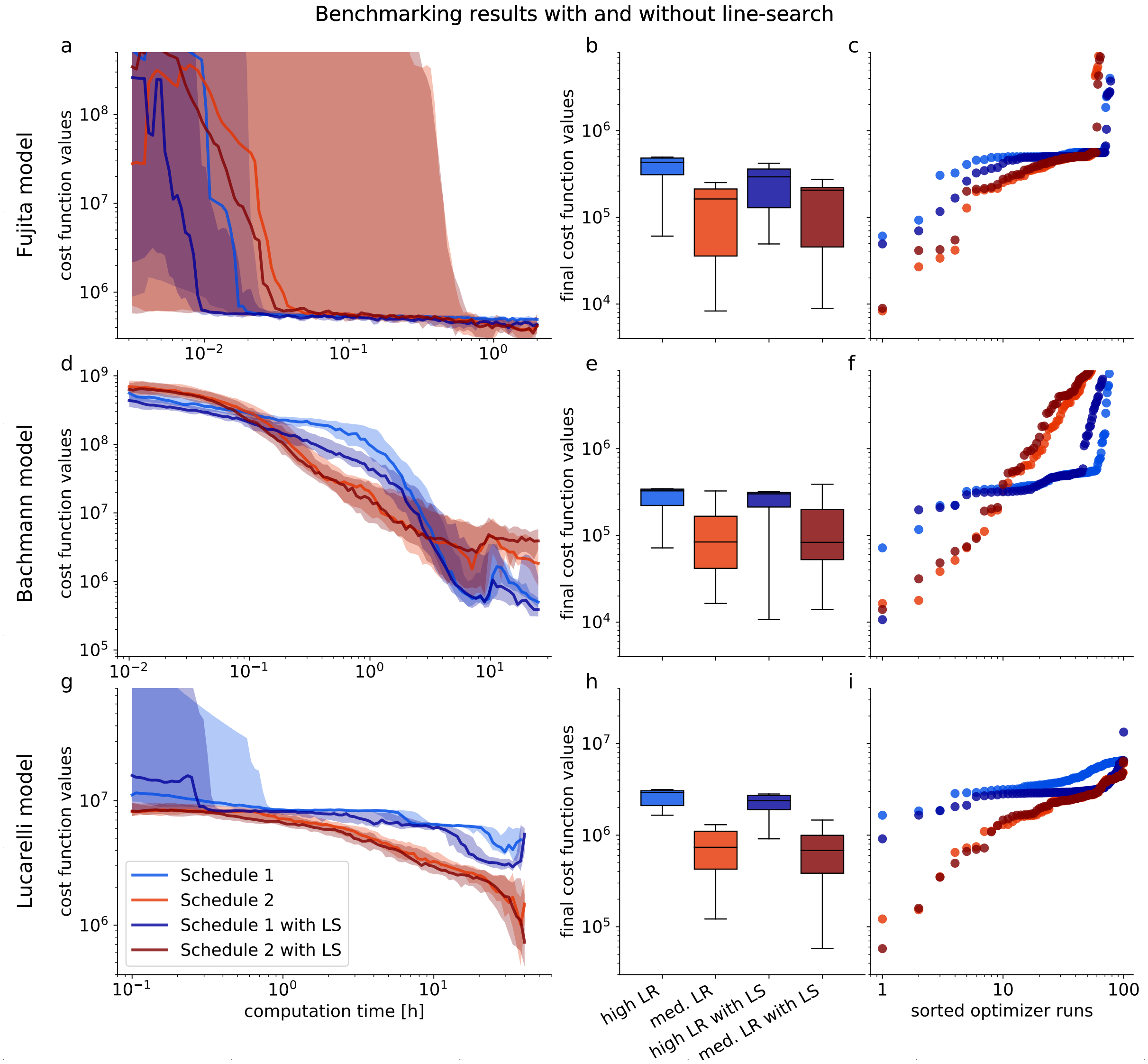

Supplementary Figure 8: Line-search has positive effect for mini-batch optimization methods at high learning rates (Schedule 1) (Supplementary to Fig. 3 of main manuscript). Optimizations at high and medium learning rates (Schedules 1 and 2) are depicted for small- to medium-scale models. Objective function values along optimization traces were extrapolated from mini-batch to the full dataset, smoothed over multiple optimization steps to filter the stochasticity of the mini-batches, and interpolated for a mesh of computation time points. Shading depicts percentiles for 10% and 40% of optimizer traces, solid lines depict percentiles for 20%. Final objective function values for box and waterfall plots were evaluated at final point of optimization trajectory. **a** Traces of objective function values during optimization for Fujita model. **b** Box plots of the 10 best final objective function values for Fujita model. **c** Waterfall plot of all final objective function values for Fujita model. **d** Traces of objective function values during optimization for Bachmann model. **e** Box plots of the 10 best final objective function values for Bachmann model. **f** Waterfall plot of all final objective function values for Bachmann model. **g** Traces of objective function values during optimization for Lucarelli model. **h** Box plots of the 10 best final objective function values for Lucarelli model. **i** Waterfall plot of all final objective function values for Lucarelli model.

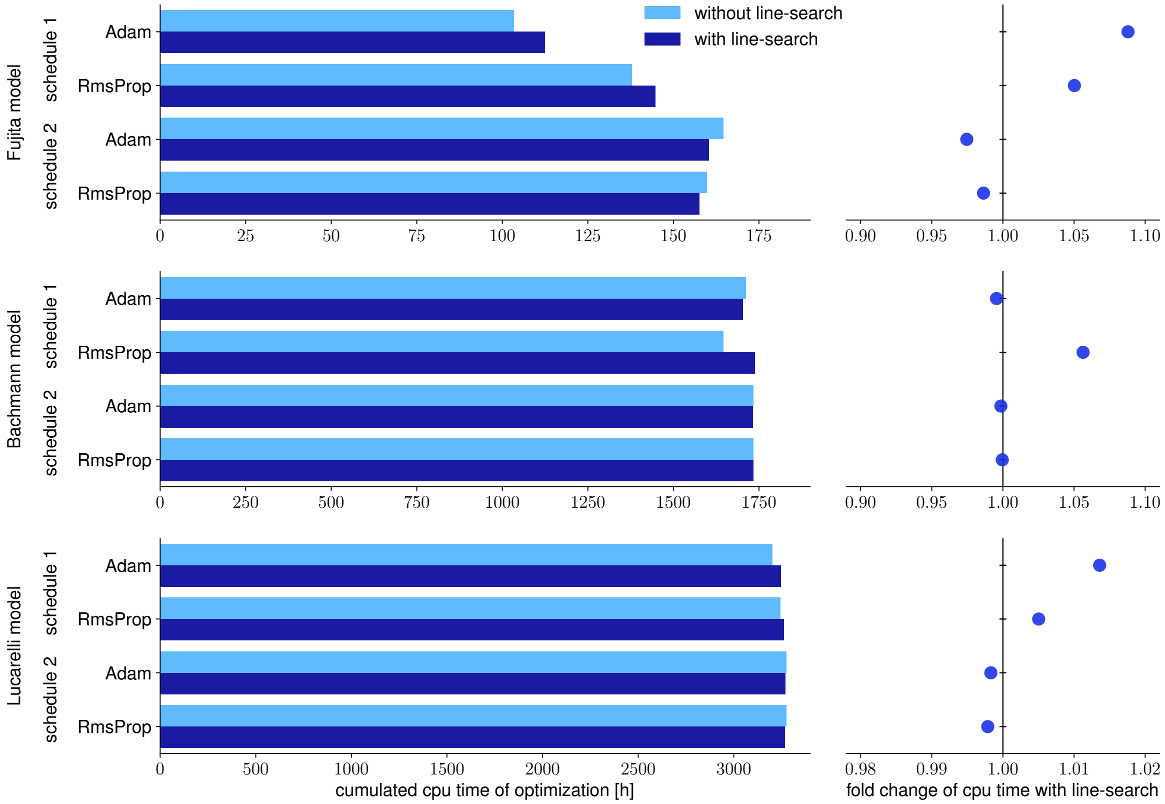

Supplementary Figure 9: Line-search has only a mild effect on the total computation time in mini-batch optimization. Total cpu times for the optimization with the two most successful learning rate Schedules (Schedule 1, high learning rate and Schedule 2, medium learning rate) with rescue interceptor and with and without line-search, depicted as bar plots (left hand side) and cpu time ratios (right hand side), for the three small- to medium-scale example models Fujita (upper panel), Bachmann (middle panel), and Lucarelli (lower panel).

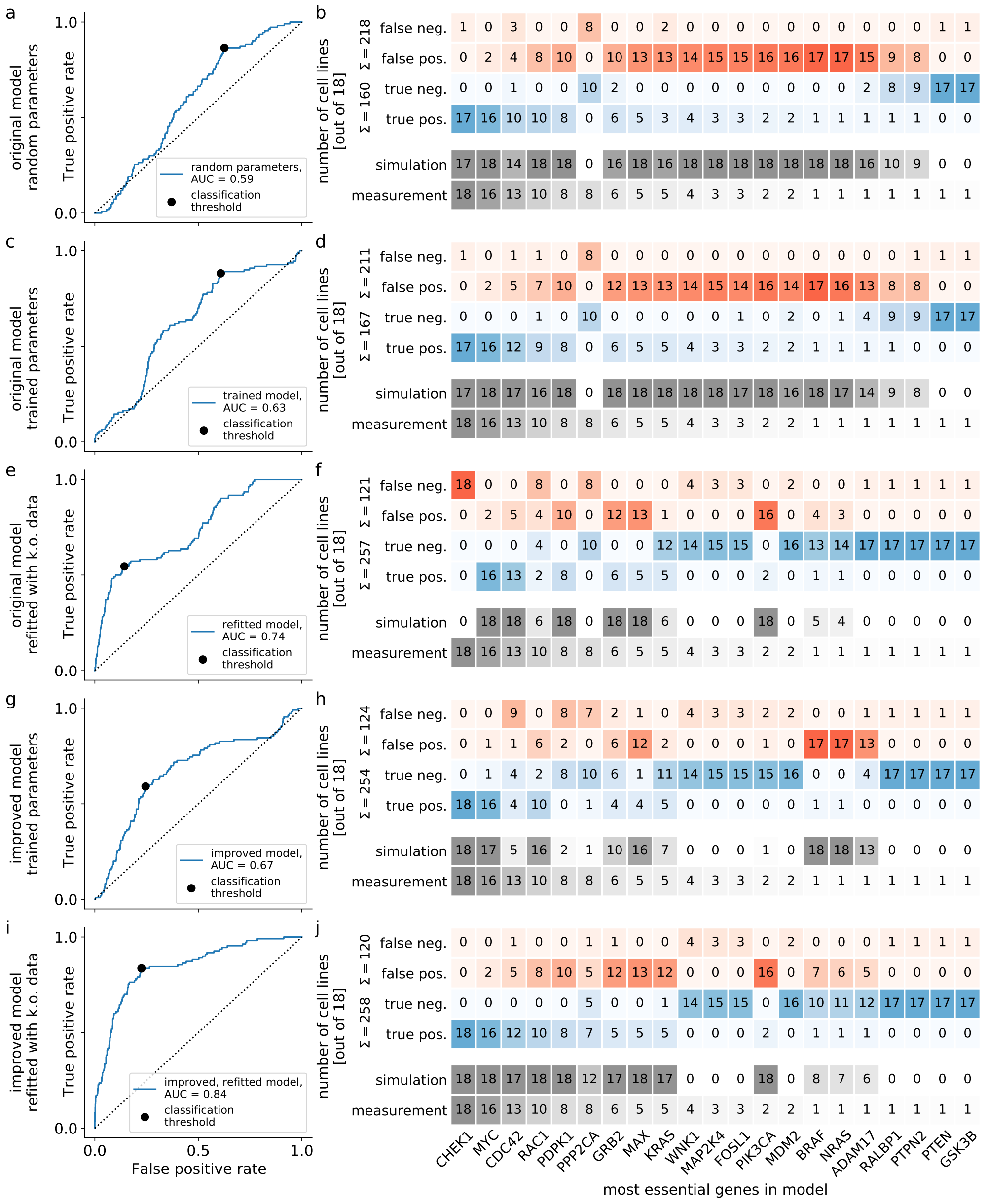

Supplementary Figure 10: In-silico knockout simulations of the large-scale cancer model for different parameter sets, compared against experimental data from Behan et al, 2019 (Supplementary to Fig. 5 of main manuscript). **a** ROC-curve of gene essentiality for the original model, using random parameters, with area under ROC and classification threshold depicted. **b** Measurement, prediction, and confusion matrix for essential genes for the original model with random parameters. Numbers indicate how often a gene was found to be essential in experimental data and in-silico knockout predictions for the 18 cell lines, sums show the number of true and false predictions over essential genes. **c - d** Analogue to subfigures a - b, for the original model, trained on drug response data. **e - f** Analogue to subfigures a - b, for the original model, trained on drug response data and knockout data. **g - h** Analogue to subfigures a - b, for the model with improved proliferation readout, trained on drug response data. **i - j** Analogue to subfigures a - b, for the model with improved proliferation readout, trained on drug response data and knockout data.

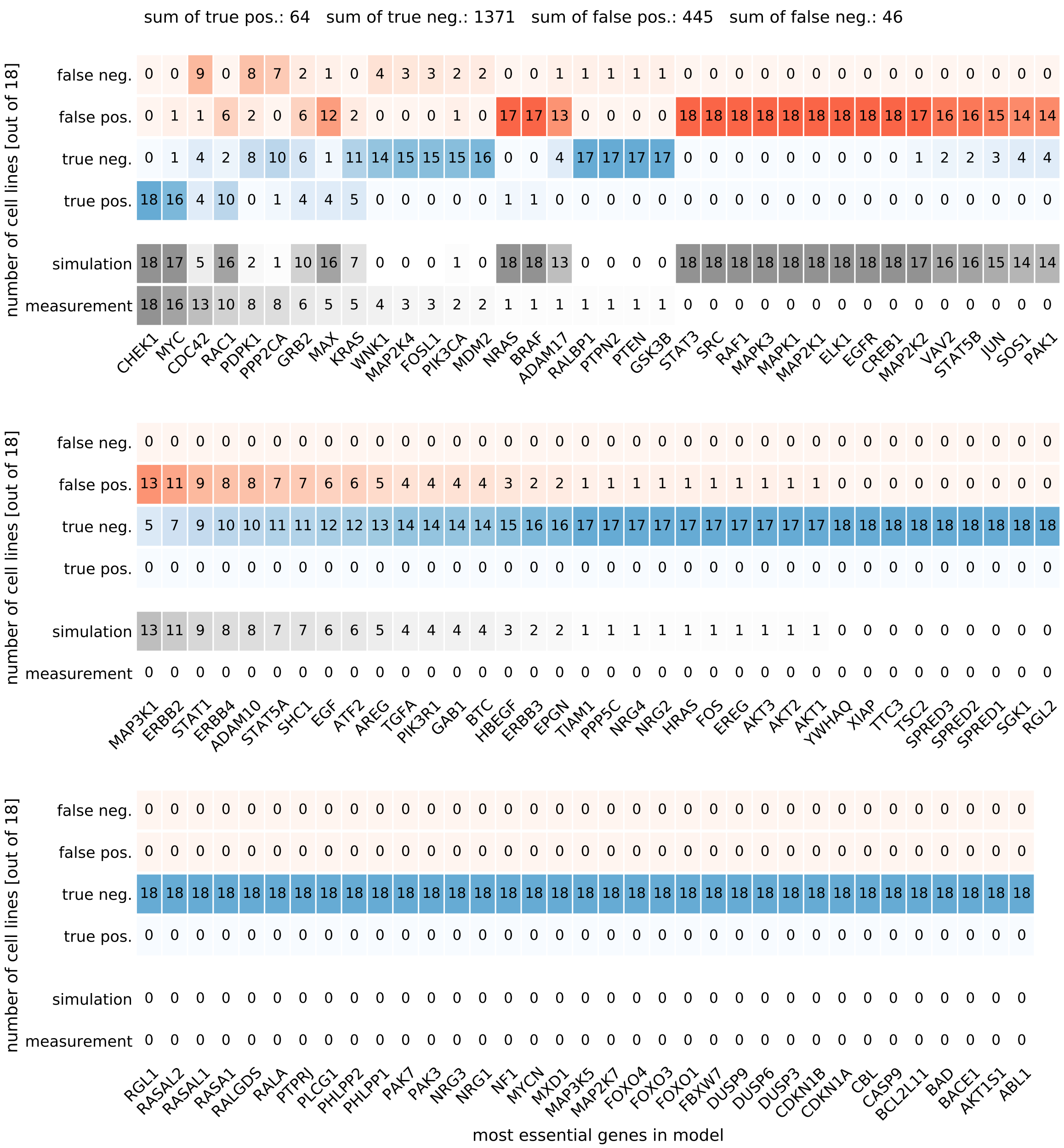

Supplementary Figure 11: In-silico knockout predictions for the refined large-scale cancer model, compared against experimental data from Behan et al, 2019 (Supplementary to Fig. 5 of main manuscript). Measurement, prediction, and confusion matrix for all genes implemented in the large-scale cancer model, after model refinement and calibration on drug response data. Numbers indicate how often a gene was found to be essential in experimental data and in-silico knockout predictions for the 18 cell lines, sums show the number of true and false predictions over essential genes.

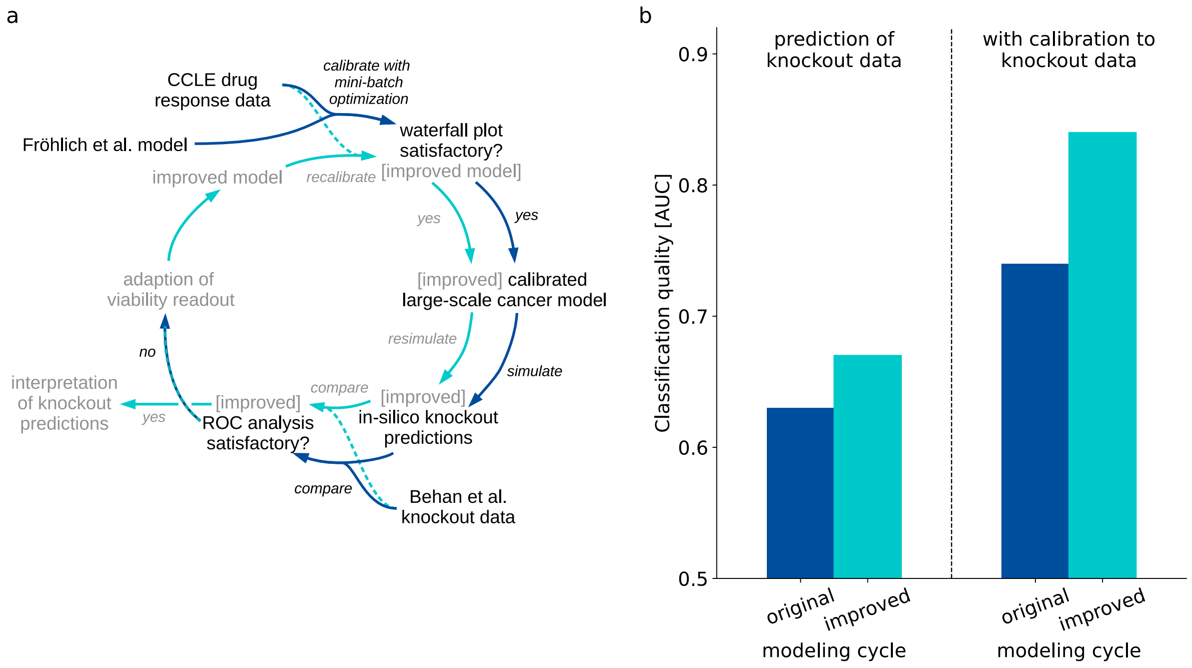

Supplementary Figure 12: Systems biology modeling cycle, illustrated for the large-scale cancer signaling ODE model (Supplementary to Fig. 5 of main manuscript). **a** Classical modeling cycle, consisting of model creation, model calibration, performing predictions from the calibrated model and assessing the prediction quality based on validation data. If the prediction quality is satisfactory, the modeler can continue with quantifying the uncertainty of the model predictions and with their biological interpretation. Otherwise, the model is usually refined and the cycle is restarted. For the Fröhlich et al. model, the viability readout was adapted in the second iteration. **b** Model performance on gene knockout data from Behan et al. before (dark blue) and after (cyan) model refinement. In a first step, both models were calibrated on drug response data from CCLE only, and the in-silico knockouts predicted from the trained models. In a second step, the models were fitted to CCLE and knockout data simultaneously, hence the in-silico knockouts are fitted.

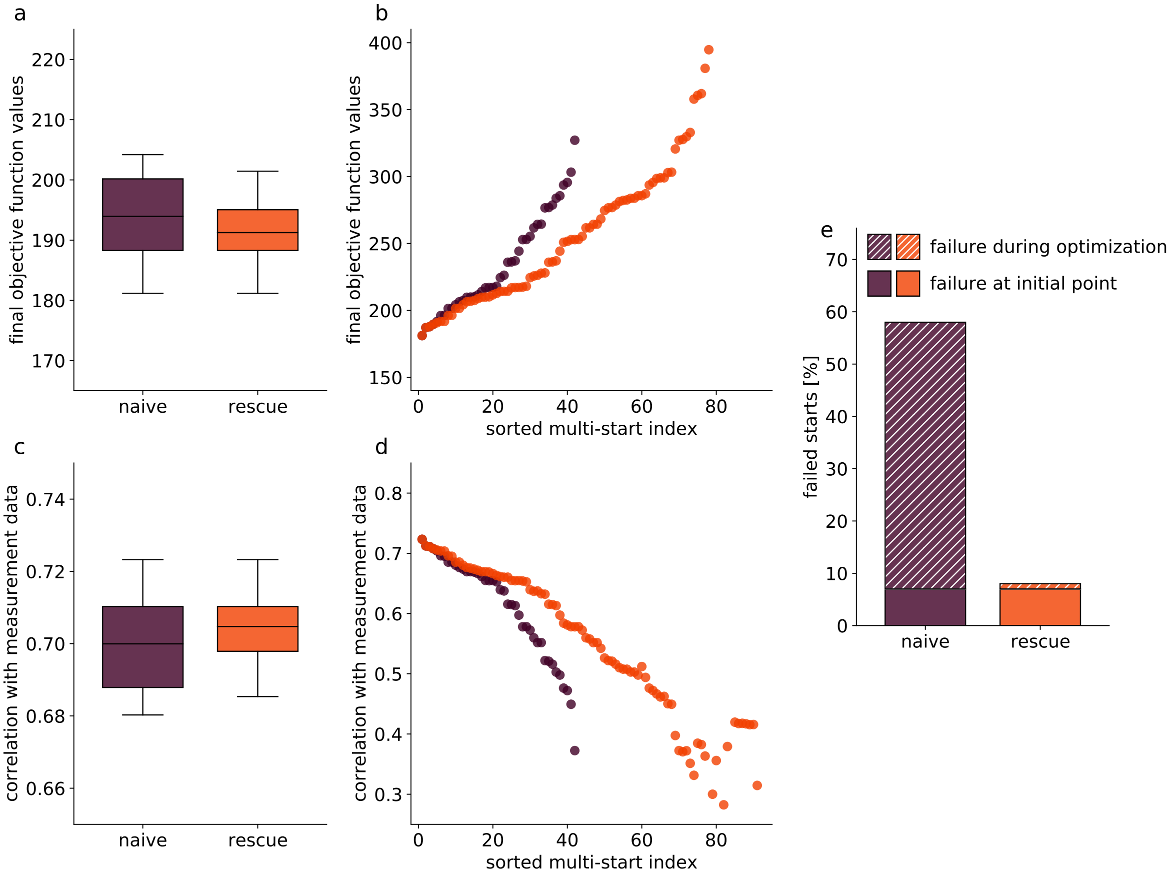

Supplementary Figure 13: Rescue interceptor reduces failure rate for mini-batch optimization on large-scale ODE model (Supplementary to Fig. 6 of main manuscript). Optimization results compared for Adam, mini-batch size 100, at low, decreasing learning rate, with and without rescue interceptor. **a** Box plots with final objective function values of the 10 best optimization results. **b** Waterfall plot with final objective function values of optimization results. **c** Box plots with values of correlation between model simulation and measurement data of the 10 best optimization results. **d** Waterfall plot with values of correlation between model simulation and measurement data of optimization results. **e** Bar plot of optimization run failure due to non-integrability of the ODE with and without rescue functionality, indicating failure at initial point of optimization.

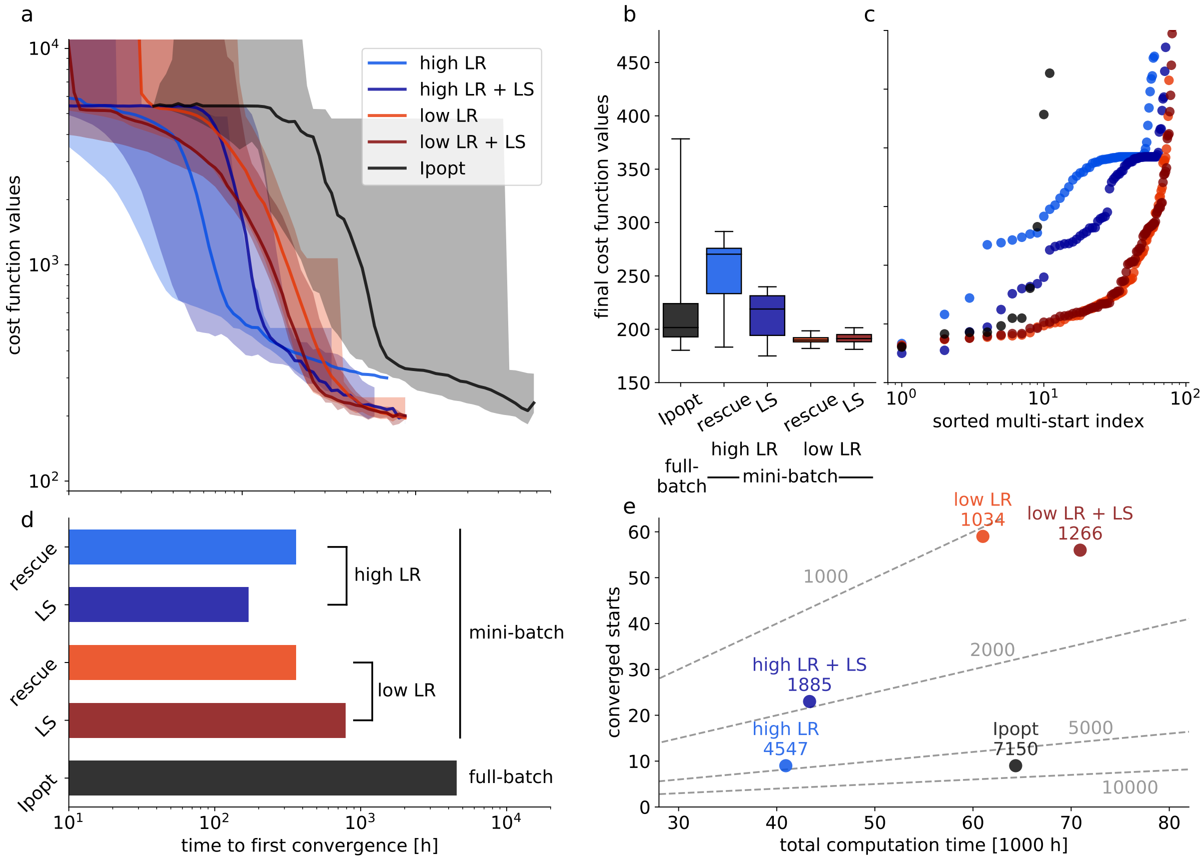

Supplementary Figure 14: Line-search markedly improves optimization performance for medium learning rates, without substantially obstructing optimization performance for low learning rates on large-scale application example (Supplementary to Fig. 6 of main manuscript). Different metrics of optimization performance are depicted, mini-batch optimization with Adam at mini-batch size 100 was benchmarked for different learning rates with and without additional line-search against full-batch optimizer Ipopt. **a** Traces of objective function values during optimization for the ten best starts plotted against computation time. Objective function values along optimization traces were extrapolated from mini-batch to the full dataset, smoothed over multiple optimization steps to filter the stochasticity of the mini-batches, and interpolated for a mesh of computation time points. Shading depicts the full envelope of the ten best optimizer traces, solid lines depict the medians. **b** Box plots of final objective function values of ten best optimization runs, evaluated at final point of optimization. **c** Waterfall plots of final objective function values of all optimization runs, evaluated at final point of optimization. **d** Bar plots of computation time, until first start converged. **e** Visualization of converged starts against accumulated total computation time showing the computation time per converged start (the lower the value, the better the optimization performance).

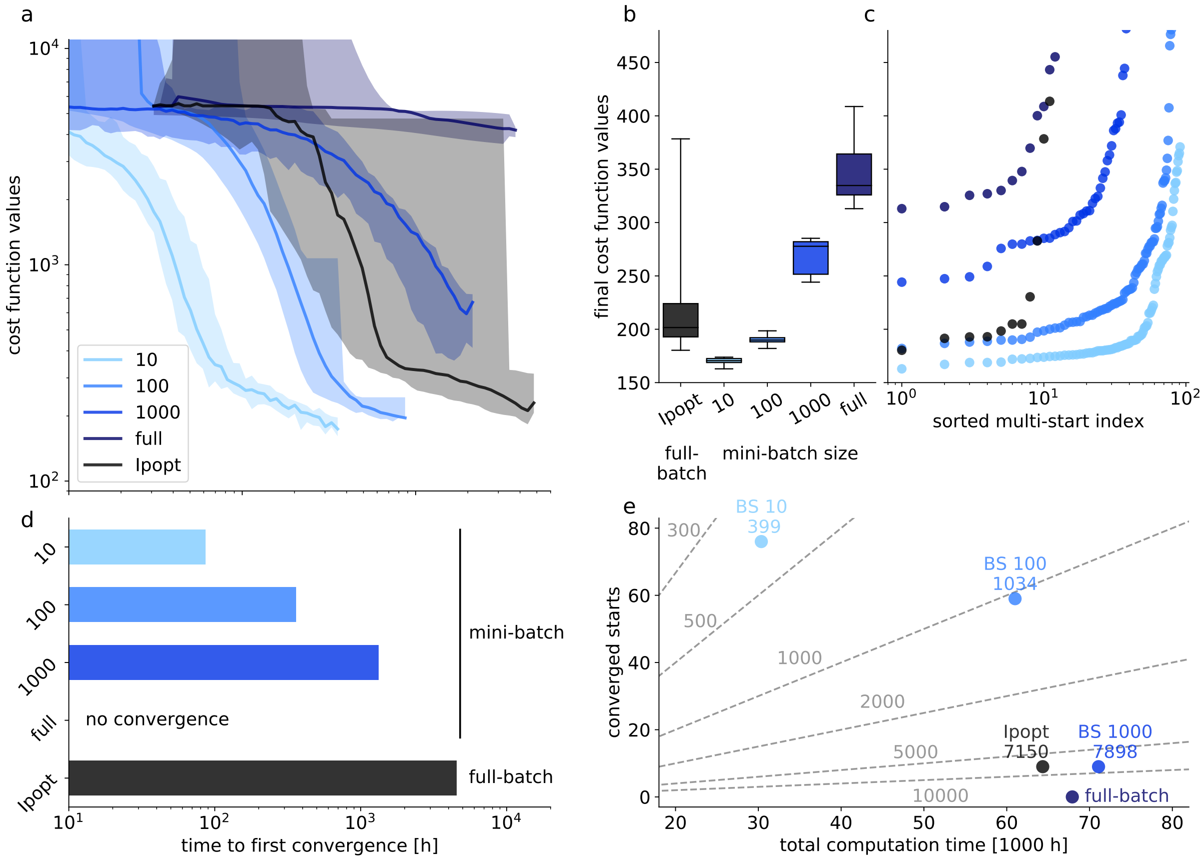

Supplementary Figure 15: Small mini-batch sizes improve optimization performance for mini-batch optimization on large-scale application example (Supplementary to Fig. 6 of main manuscript). Different metrics of optimization performance are depicted, mini-batch optimization with Adam at mini-batch size 100 was benchmarked for different mini-batch sizes at low learning rates without additional line-search against full-batch optimizer Ipopt. **a** Traces of objective function values during optimization for the ten best starts plotted against computation time. Objective function values along optimization traces were extrapolated from mini-batch to the full dataset, smoothed over multiple optimization steps to filter the stochasticity of the mini-batches, and interpolated for a mesh of computation time points. Shading depicts the full envelope of the ten best optimizer traces, solid lines depict the medians. **b** Box plots of final objective function values of ten best optimization runs, evaluated at final point of optimization. **c** Waterfall plots of final objective function values of all optimization runs, evaluated at final point of optimization. **d** Bar plots of computation time, until first start converged. **e** Visualization of converged starts against accumulated total computation time showing the computation time per converged start (the lower the value, the better the optimization performance).

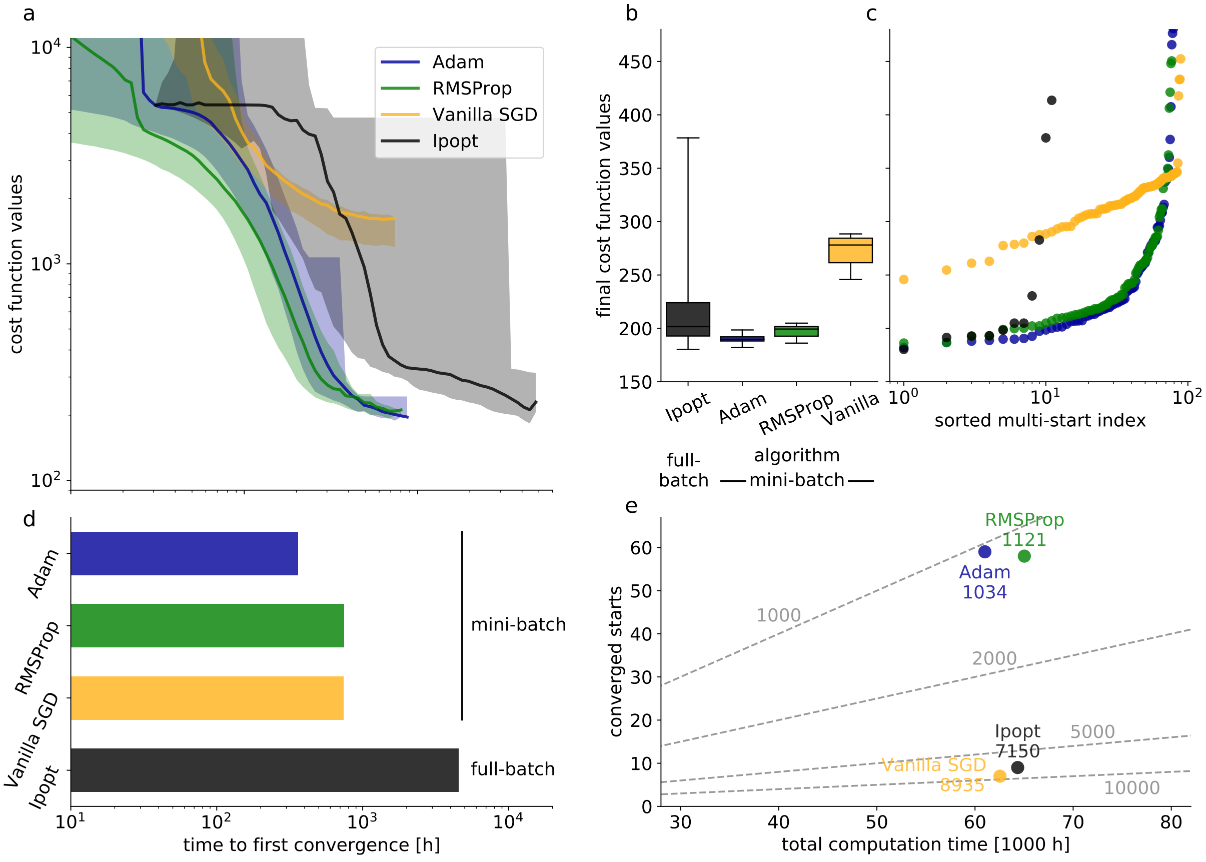

Supplementary Figure 16: Adam and RMSProp algorithm showing comparable optimization performance on large-scale application example (Supplementary to Fig. 6 of main manuscript). Different metrics of optimization performance are depicted, mini-batch optimization at mini-batch size 100 and was benchmarked for different mini-batch algorithms at low learning rates against full-batch optimizer Ipopt. To ensure similar step-sizes in optimization for all optimizers, we used low learning rates for RMSProp and Adam and a high learning rate (10^0 to 10^{-3}) for Vanilla SGD. **a** Traces of objective function values during optimization for the ten best starts plotted against computation time. Objective function values along optimization traces were extrapolated from mini-batch to the full dataset, smoothed over multiple optimization steps to filter the stochasticity of the mini-batches, and interpolated for a mesh of computation time points. Shading depicts the full envelope of the ten best optimizer traces, solid lines depict the medians. **b** Box plots of final objective function values of ten best optimization runs, evaluated at final point of optimization. **c** Waterfall plots of final objective function values of all optimization runs, evaluated at final point of optimization. **d** Bar plots of computation time, until first start converged. **e** Visualization of converged starts against accumulated total computation time showing the computation time per converged start (the lower the value, the better the optimization performance).

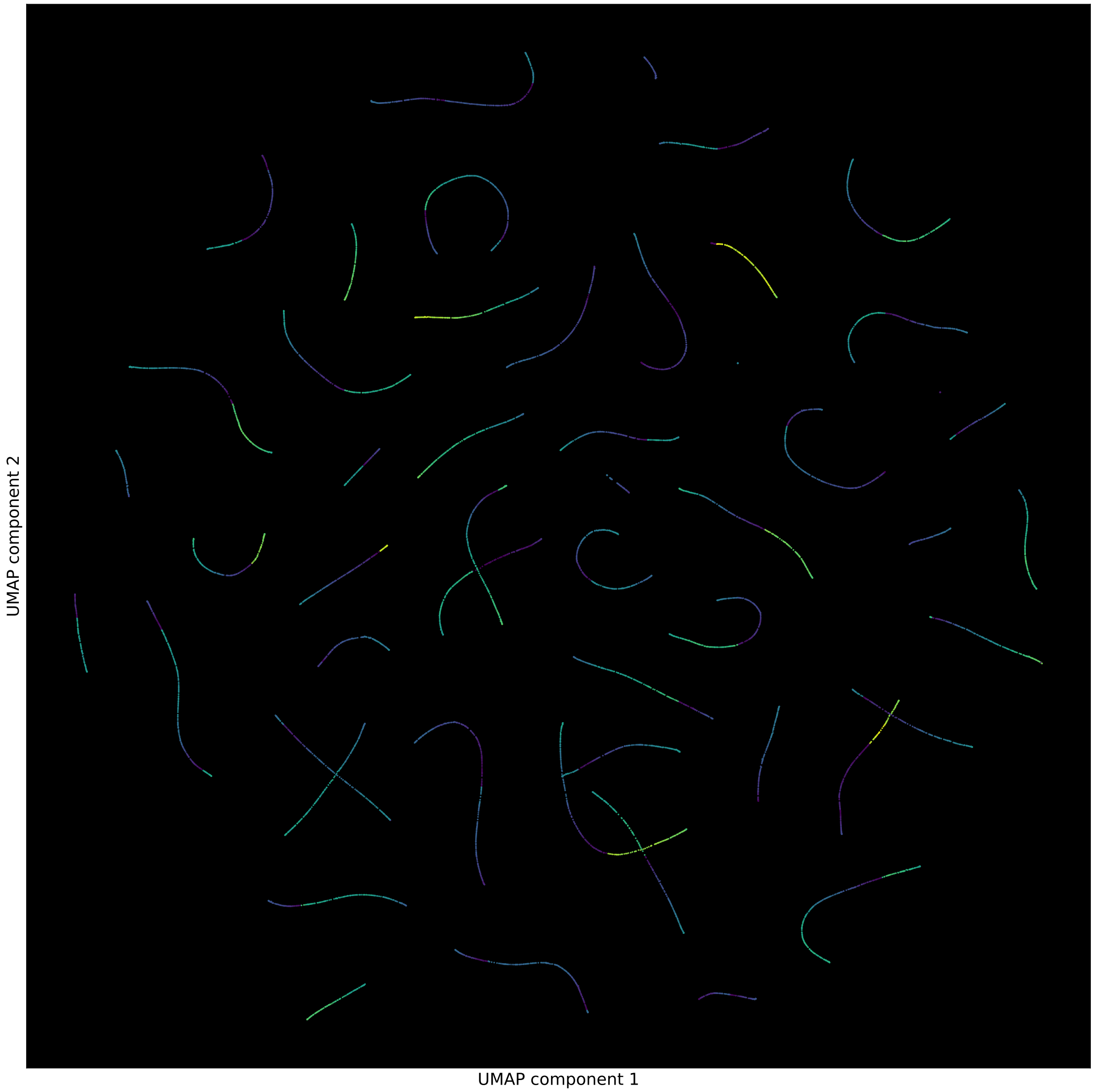

Supplementary Figure 17: Visualization of UMAP dimension reduction (19) (first two components shown) for the large parameter ensemble, created from mini-batch optimization with mini-batch size 10, on the original large-scale cancer model. Brighter colors indicate better objective function values. The location of the end point of the local optimization runs – overall 52 optimization runs are represented in the ensemble – shows no clear pattern. Also the optimization runs themselves do not seem to be aligned. Hence, we assume there was no mixing of the different optimization runs and exploration of parameter space was not yet fully satisfactory.
